# IL-9/STAT3/fatty acid oxidation–mediated lipid peroxidation contributes to Tc9 cell longevity and enhanced antitumor activity

**DOI:** 10.1172/JCI153247

**Published:** 2022-04-01

**Authors:** Liuling Xiao, Xingzhe Ma, Lingqun Ye, Pan Su, Wei Xiong, Enguang Bi, Qiang Wang, Miao Xian, Maojie Yang, Jianfei Qian, Qing Yi

**Affiliations:** Center for Translational Research in Hematologic Malignancies, Houston Methodist Cancer Center/Houston Methodist Research Institute, Houston Methodist, Houston, Texas, USA.

**Keywords:** Immunology, Metabolism, Cancer immunotherapy, Fatty acid oxidation, T cells

## Abstract

CD8^+^ T cell longevity regulated by metabolic activity plays important roles in cancer immunotherapy. Although in vitro–polarized, transferred IL-9–secreting CD8^+^ Tc9 (cytotoxic T lymphocyte subset 9) cells exert greater persistence and antitumor efficacy than Tc1 cells, the underlying mechanism remains unclear. Here, we show that tumor-infiltrating Tc9 cells display significantly lower lipid peroxidation than Tc1 cells in several mouse models, which is strongly correlated with their persistence. Using RNA-sequence and functional validation, we found that Tc9 cells exhibited unique lipid metabolic programs. Tc9 cell–derived IL-9 activated STAT3, upregulated fatty acid oxidation and mitochondrial activity, and rendered Tc9 cells with reduced lipid peroxidation and resistance to tumor- or ROS-induced ferroptosis in the tumor microenvironment. IL-9 signaling deficiency, inhibiting STAT3, or fatty acid oxidation increased lipid peroxidation and ferroptosis of Tc9 cells, resulting in impaired longevity and antitumor ability. Similarly, human Tc9 cells also exhibited lower lipid peroxidation than Tc1 cells and tumor-infiltrating CD8^+^ T cells expressed lower *IL9* and higher lipid peroxidation– and ferroptosis-related genes than circulating CD8^+^ T cells in patients with melanoma. This study indicates that lipid peroxidation regulates Tc9 cell longevity and antitumor effects via the IL-9/STAT3/fatty acid oxidation pathway and regulating T cell lipid peroxidation can be used to enhance T cell–based immunotherapy in human cancer.

## Graphical abstract

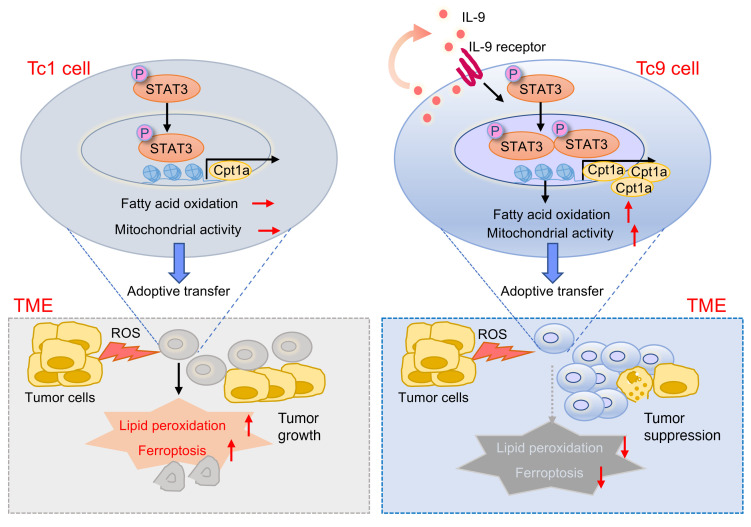

## Introduction

Adoptive cell transfer (ACT) is a highly personalized immunotherapy that utilizes ex vivo expansion and reinfusion of tumor-bearing-host lymphocytes to treat established cancers and has achieved great clinical success (1–3). A recent study showed that the objective response rates in clinical trials of ACT combined with lymphodepletion and IL-2 were 49% to 72% in metastatic melanoma. However, the complete response rates were only 8% to 28% (4). Current strategies for improving the effectiveness of transferred CD8^+^ T cells include polarizing naive CD8^+^ T cells with different cytokines (5–8), reinfusing tumor-specific CD8^+^ T cells at various differentiation states (9, 10), and developing T cell receptor (TCR) or chimeric antigen receptor (CAR) genetically engineered T cells (11, 12). Among these, developing a CD8^+^ T cell subset with better persistence will greatly improve the therapeutic efficacy of ACT therapy in human cancer.

CD8^+^ T cells were originally regarded as uniformly cytotoxic cells that secret IFN-γ and cytolytic granules to kill infected or tumorigenic cells. However, accumulating evidence demonstrates that CD8^+^ T cells can be classified into Tc1, Tc2, Tc9, Tc17, and Tc22 subsets (13, 14). Each CD8^+^ T subset is functionally distinct and has different roles in tumor immune responses. As a typical cytotoxic CD8^+^ T subset, Tc1 cells play an important role in clearance of pathogens and tumors. However, IL-2–induced, T-bet^hi^ Tc1 cells have a short lifespan in the suppressive tumor microenvironment (TME) after ACT (15, 16), and our previous studies showed that adoptively transferred tumor-specific Tc9 cells elicit a stronger antitumor response against advanced tumors than Tc1 cells (5, 17). After adoptive transfer, Tc9 cells convert to IFN-γ– and granzyme B–secreting cytotoxic effector T cells while maintaining less exhausted and longer survival features as compared with Tc1 cells. However, it remains unclear how Tc9 cells persist longer than Tc1 cells in the TME.

The TME, which is commonly acidic, hypoxic, depleted of critical nutrients, and rich in immunosuppressive compounds (18, 19), is deleterious for the longevity of adoptively transferred T cells. Many methods have been explored to increase the persistence of T cells (20–22). Growing evidence has indicated that the cellular metabolic state is a critical determinant for CD8^+^ T cell longevity and antitumor activity (23–25). Our previous studies showed that the TME is enriched with cholesterol, which induces CD8^+^ T cell CD36 expression and exhaustion. Elevated CD36 expression on CD8^+^ T cells dampens their antitumor function by inducing T cell ferroptosis in the TME (16, 26). Ferroptosis is a form of cell death and is induced by excessive lipid peroxidation in cells (27). As we previously reported that Tc9 cells have lower cholesterol levels than Tc1 cells, we wondered whether Tc9 cells have a lower level of ferroptosis than Tc1 cells and whether this contributes to the better persistence and antitumor effect of Tc9 cells in the TME.

In this study, we observed that tumor-infiltrating Tc9 cells displayed significantly lower lipid peroxidation and ferroptosis than Tc1 cells after transfer into tumor-bearing mice, which contributed to their longevity and antitumor efficacy. Mechanistic studies revealed that the IL-9/STAT3/fatty acid oxidation pathway rendered Tc9 cells with reduced lipid peroxidation and resistance to tumor- or ROS-induced ferroptosis in the TME.

## Results

### Persistence of adoptively transferred T cells in the TME is negatively correlated to their lipid peroxidation levels.

**Figure 1:**
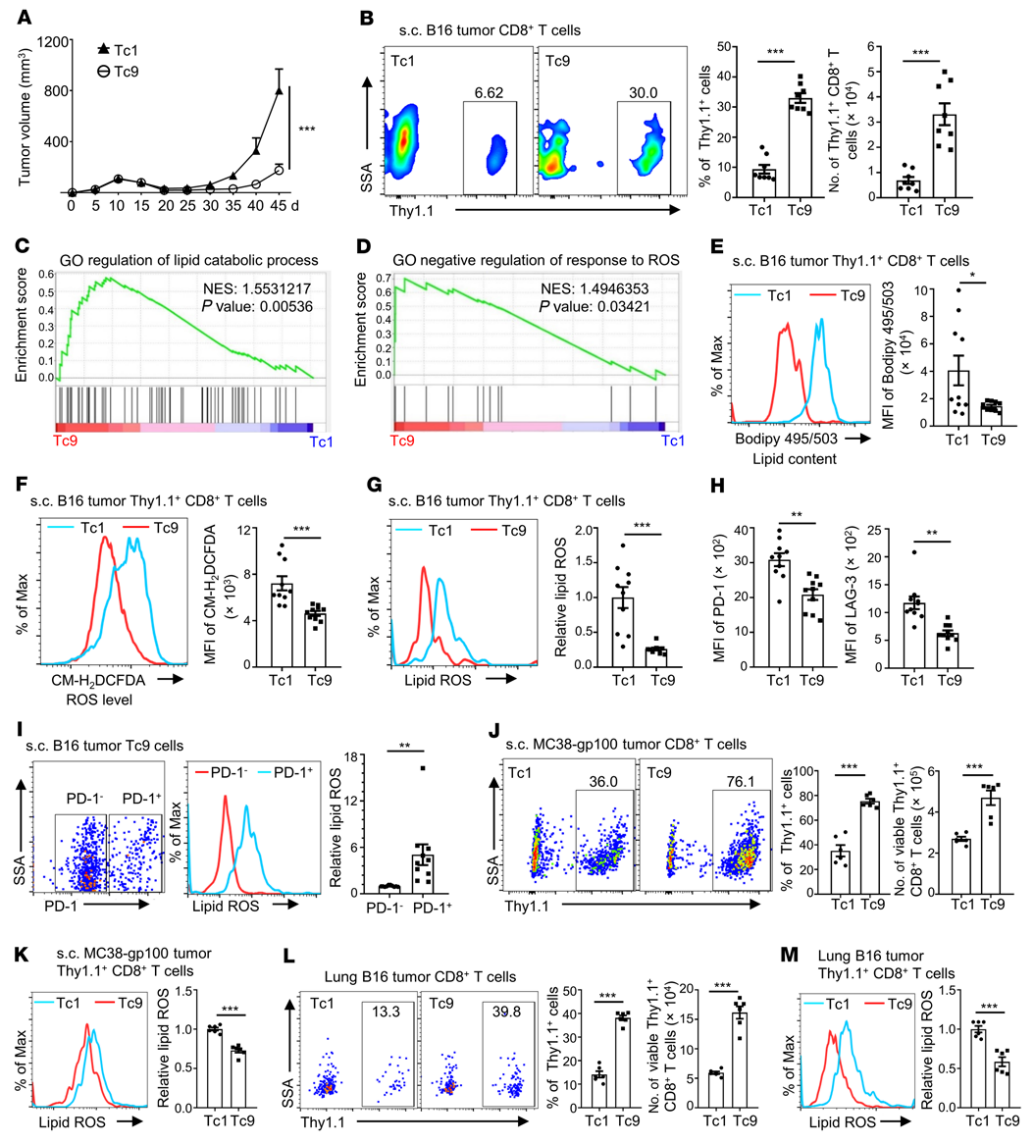
Persistence of adoptively transferred CD8^+^ T cells in the TME is negatively correlated with their lipid peroxidation. (**A** and **B**) Thy1.1^+^ Pmel-1 Tc1 or Tc9 cells were i.v. injected into Thy1.2^+^ B6 mice bearing 10-day s.c. B16 tumors with adjuvant treatments (CTX, dendritic cells, and rhIL-2). Tumor growth curve (*n =* 5), Thy1.1^+^ percentages in CD8^+^ T cells, and Thy1.1^+^ CD8^+^ T cell numbers in tumors on day 45 after tumor injection (*n =* 8). (**C** and **D**) GSEA of indicated gene sets on day 24 after tumor injection. GO, Gene Ontology; NES, normalized enrichment score. (**E**) Lipid content (BODIPY 495/503 staining), (**F**) total ROS level (CM-H_2_DCFDA staining), (**G**) lipid ROS, and (**H**) PD-1 and LAG-3 expression of transferred Tc1 and Tc9 cells on day 45 after tumor injection (*n =* 10, two pooled independent experiments). (**I**) Tumor-infiltrating Tc9 cells were divided into PD-1^+^ and PD-1^–^ groups and analyzed for the level of lipid ROS (*n =* 10). (**J** and **K**) Thy1.1^+^ Pmel-1 Tc1 or Tc9 cells were i.v. injected into Thy1.2^+^ B6 mice bearing 10-day MC38-gp100 tumors with adjuvant treatments. Shown are Thy1.1^+^ percentages in CD8^+^ T cells, Thy1.1^+^ CD8^+^ T cell numbers, and relative lipid ROS levels of Tc1 and Tc9 cells in tumors on day 40 after tumor injection (*n =* 6). (**L** and **M**) Thy1.1^+^ Pmel-1 Tc1 or Tc9 cells were i.v. injected into Thy1.2^+^ B6 mice bearing 12-day lung metastatic B16 tumors. Shown are Thy1.1^+^ percentages in CD8^+^ T cells, Thy1.1^+^ CD8^+^ T cell numbers, and relative lipid ROS levels of Tc1 and Tc9 cells in tumors on day 17 after tumor injection (*n =* 6). Data are presented as mean ± SEM. Tumor-infiltrating Thy1.1^+^ CD8^+^ T cell number was normalized to 100 mg tumor tissue. *P < 0.05; **P < 0.01; ***P < 0.001 by 2-way ANOVA in **A** and unpaired, 2-tailed Student’s *t* test in the other panels.

Our previous studies showed that adoptively transferred Tc9 cells display better persistence than Tc1 cells in the TME (5, 17); however, the underlying mechanism remains unclear. To elucidate this mechanism, we treated subcutaneous (s.c.) B16 tumor–bearing Thy1.2^+^ mice with Thy1.1^+^ Pmel-1–derived Tc1 or Tc9 cells. Consistent with our previous findings (5, 17), Tc9 cells showed greater suppression of tumor growth (Figure 1A and Supplemental Figure 1A; supplemental material available online with this article; https://doi.org/10.1172/JCI153247DS1) and better persistence in tumor tissues than Tc1 cells (Figure 1B). RNA sequencing (RNA-Seq) results from tumor-infiltrating Tc1 and Tc9 cells showed that genes regulating lipid catabolic processes and inhibiting ROS production were greatly enriched in Tc9 cells compared with Tc1 cells by using Gene Set Enrichment Analysis (GSEA) (Figure 1, C and D), which suggests lower levels of lipids and ROS in Tc9 cells than in Tc1 cells. We then detected lipid content and cellular ROS level in the T cells in tumor tissues by flow cytometry and confirmed that lipid and cellular ROS levels were indeed lower in Tc9 than Tc1 cells (Figure 1, E and F), and Tc9 cells had lower ROS production but similar ROS scavenging ability compared to Tc1 cells (Supplemental Figure 1, B and C).

Lipid peroxidation is oxidative degradation of lipids and occurs when oxidants such as ROS attack lipids containing carbon-carbon double bond(s) (28). As both lipids and cellular ROS levels were lower in Tc9 cells, we hypothesized that Tc9 cells have lower lipid peroxidation than Tc1 cells. To test this hypothesis, we measured lipid ROS (lipid peroxidation sensor) levels in tumor-infiltrating Tc1 and Tc9 cells from s.c. B16 tumor–bearing mice. As expected, Tc9 cells had significantly lower lipid ROS than Tc1 cells (Figure 1G). Additionally, we found that the expression of exhaustion markers PD-1 and LAG-3 was also lower on Tc9 than Tc1 cells (Figure 1H). Compared with Tc9 cells, more Tc1 cells were PD1^+^ and these cells were enriched with an exhaustion-upregulated gene signature (Supplemental Figure 2, A and B). When we divided the transferred tumor-infiltrating Tc9 cells into PD-1^+^ and PD-1^–^ groups, PD-1^+^ Tc9 cells were found to contain more lipid ROS than PD-1^–^ T cells (Figure 1I). Moreover, we observed a higher percentage of transferred Thy1.1^+^CD8^+^ T cells in peripheral blood and spleen from Tc9 cell–treated tumor-bearing mice than those treated with Tc1 cells (Supplemental Figure 2, C and E) and less lipid ROS in circulating and splenic Tc9 cells than Tc1 cells (Supplemental Figure 2, D and F).

We used two additional tumor models to confirm the results. Similarly, compared with Tc1 cells, adoptively transferred tumor-specific Tc9 cells showed better antitumor activity (Supplemental Figure 2, G and K) and persistence in tumors and spleens (Figure 1, J and L, and Supplemental Figure 2, H, I, and L), and tumor-infiltrating and splenic Tc9 cells contained less lipid ROS (Figure 1, K and M, and Supplemental Figure 2, J and M) in both s.c. MC38-gp100 colon cancer and B16 lung metastatic models. Taken together, these results indicate that lipid peroxidation is negatively correlated with the persistence of adoptively transferred T cells in vivo.

### Reduced lipid peroxidation and ferroptosis are required for the longevity of Tc9 cells in vivo.

**Figure 2:**
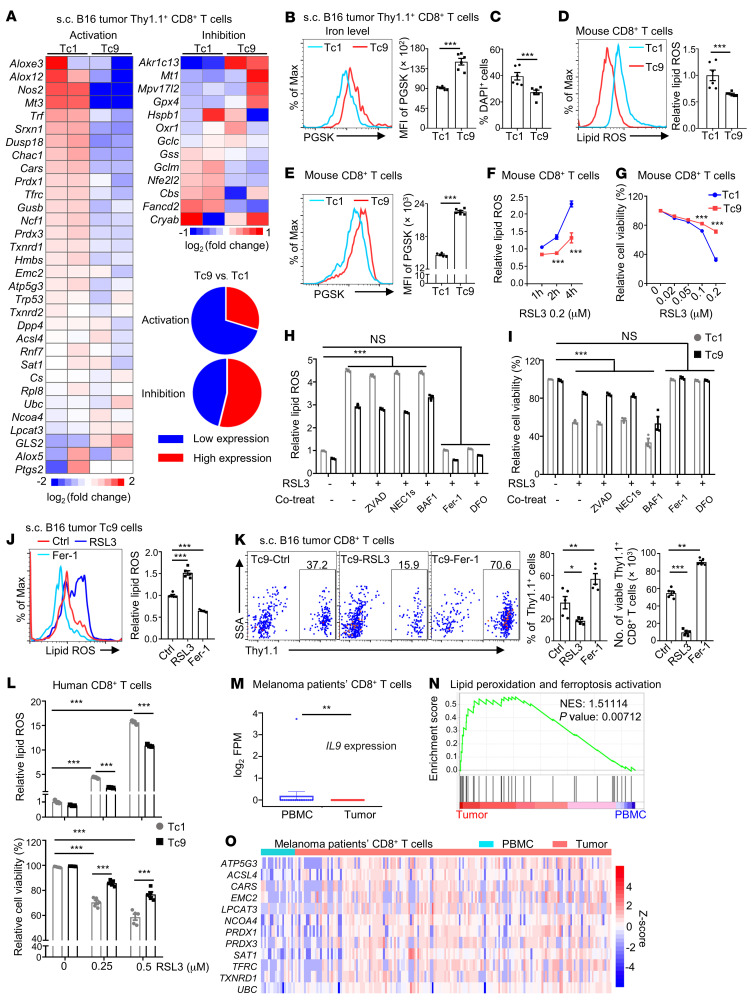
Reduced lipid peroxidation and ferroptosis are required for the longevity of Tc9 cells in vivo. (**A**–**C**) Thy1.1^+^ Pmel-1 Tc1 or Tc9 cells were i.v. injected into B16 tumor–bearing Thy1.2^+^ B6 mice with adjuvant treatments. Heatmap analysis of lipid peroxidation–related and ferroptosis activation– or inhibition–related genes between sorted Tc1 and Tc9 cells from RNA sequencing in **A**. Iron level and cell death of transferred tumor-infiltrating Tc1 and Tc9 cells are shown in **B** and **C**, respectively (*n =* 6). (**D**) Relative lipid ROS and (**E**) iron level from in vitro–polarized mouse Tc1 and Tc9 cells (*n =* 5–6). (**F**–**I**) Relative lipid ROS and cell viability in polarized mouse Tc1 and Tc9 cells treated with RSL3 alone or in combination with 50 mM ZVAD-FMK (ZVAD), 10 mM necrostatin-1s (NEC1s), 0.1 μM bafilomycin A1 (BAF1), 5 μM ferrostatin-1 (Fer-1), or 10 mM deferoxamine (DFO). (**J** and **K**) Thy1.1^+^ Pmel-1 Tc9 cells treated with RSL3 (0.05 μM) or Fer-1 (5 μM) before injection into B16 tumor–bearing Thy1.2^+^ B6 mice with adjuvant treatments. Relative lipid ROS, Thy1.1^+^ percentages in CD8^+^ T cells, and Thy1.1^+^ CD8^+^ T cell numbers in tumors on day 40 after tumor injection (*n =* 5). (**L**) Relative lipid ROS and cell viability in polarized human Tc1 and Tc9 cells treated with RSL3 (*n =* 3–5). (**M**) *IL9* expression in human CD8^+^ T cells from peripheral blood (*n =* 19) and tumors (*n =* 176) by analyzing published data. (**N**) GSEA and (**O**) heatmap analysis of indicated genes in CD8^+^ T cell from peripheral blood and tumors in **M**. NES, normalized enrichment score. Data are presented as mean ± SEM. *P < 0.05; **P < 0.01; ***P < 0.001 by 2-way ANOVA in **F**–**I** and **L**, 1-way ANOVA followed by Dunnett’s test in **J** and **K**, and unpaired, 2-tailed Student’s *t* test in the other panels.

Ferroptosis is a type of regulated cell death and is categorized as an iron-dependent accumulation of lipid peroxidation downstream of metabolic dysfunction (27, 29). As we detected lower lipid peroxidation in adoptively transferred Tc9 than Tc1 cells (Figure 1), we explored whether there was reduced ferroptosis in Tc9 compared with Tc1 cells. First, we analyzed RNA-Seq data of tumor-infiltrating Tc1 and Tc9 cells from s.c. B16 tumors and observed that Tc9 cells had lower expression of genes associated with lipid peroxidation and ferroptosis activation, such as *Atp5g3* and *Cars*, whereas the expression of genes associated with ferroptosis inhibition did not change significantly as compared to Tc1 cells (Figure 2A). In addition, adoptively transferred Tc9 cells had lower iron levels (higher PGSK intensity indicating lower iron level) and less cell death than Tc1 cells in tumors (Figure 2, B and C). Iron imported by transferrin and the transferrin receptor from the extracellular environment is required for the accumulation of lipid peroxides and induction of ferroptosis (30, 31). RNA-Seq results showed that the expression of transferrin (*Trf*) and transferrin receptor (*Tfrc*) was lower in Tc9 compared with Tc1 cells (Figure 2A), which was validated by real-time PCR (RT-PCR) (Supplemental Figure 3A). Interestingly, in vitro–polarized Tc9 cells had lower levels of lipid ROS (Figure 2D and Supplemental Figure 3B) and iron (Figure 2E and Supplemental Figure 3C) than Tc1 cells after activation with anti-CD3/anti-CD28 antibodies or Hgp100 peptide. When we reactivated these cells with anti-CD3/anti-CD28 antibodies, Tc9 cells again displayed less lipid ROS and less cell death than Tc1 cells (Supplemental Figure 3, D and E). Second, the ferroptosis inducers 1*S*,3*R*-RSL3 (RSL3) and FIN56 had significantly reduced abilities to induce Tc9 cells to produce lipid ROS (Figure 2F) and undergo cell death (Figure 2G and Supplemental Figure 3F) than Tc1 cells. Third, the ferroptosis inhibitor ferrostatin-1 (Fer-1) or iron chelator deferoxamine (DFO) rescued RSL3-induced lipid peroxidation and cell death in Tc1 and Tc9 cells, whereas inhibitors of apoptosis (Z-DEVD-FMK), necroptosis (necrostatin-1s), or autophagy (bafilomycin A1) had no effect on lipid ROS production and cell death in these T cells (Figure 2, H and I). Taken together, these results indicate that Tc9 cells are more resistant to lipid peroxidation and ferroptosis induction than Tc1 cells.

To confirm that reduced ferroptosis is required for enhanced persistence of adoptively transferred Tc9 cells in tumor tissues, we treated Thy1.1^+^ Tc9 cells with RSL3 (ferroptosis inducer) or Fer-1 (ferroptosis inhibitor) before transfer. After transfer, mice injected with Fer-1–treated Tc9 cells had significantly higher percentages and numbers of Tc9 cells in tumor tissues and blood, and these T cells had a significantly lower levels of lipid ROS than control T cells. In contrast, mice injected with RSL3-treated Tc9 cells had significantly lower percentages and numbers of Tc9 cells in tumor tissues and blood, and these Tc9 cells had a higher level of lipid ROS than control Tc9 cells (Figure 2, J and K, and Supplemental Figure 3, G and H). Similar results were also obtained with RSL3- or Fer-1–treated Tc1 cells (Supplemental Figure 3, I and J). Thus, these results clearly demonstrated that reduced lipid peroxidation and ferroptosis are required for the persistence of Tc9 cells in vivo.

### Human Tc9 cells exhibit decreased lipid peroxidation and IL-9 is negatively correlated to lipid peroxidation– and ferroptosis-related genes in human melanoma CD8^+^ T cells.

A similar phenomenon was observed in human Tc9 cells. While RSL3 induced human Tc1 cell ferroptosis in a dose-dependent manner, human Tc9 cells were more resistant to RSL3-induced lipid peroxidation and ferroptosis (Figure 2L). To determine whether human CD8^+^ T cells undergo ferroptosis in tumor tissues, we analyzed published single-cell sequencing data from patients with melanoma (32). Peripheral blood CD8^+^ T cells had high expression of *IL9* (Figure 2M) and low expression of gene sets (29, 33) associated with lipid peroxidation and ferroptosis activation (Figure 2N and Supplemental Table 1), while tumor-infiltrating CD8^+^ T cells showed the opposite trend. Some of the representative genes are listed by heatmap (Figure 2O). Additionally, analysis of The Cancer Genome Atlas (TCGA) database (34, 35) further indicated that patients with high *IL9* expression showed a trend for better overall survival, although not statistically significant, compared with those with low *IL9* expression in tumor tissues with high *CD8*^+^ infiltration in human melanoma (skin cutaneous melanoma), colon cancer, and breast cancer (Supplemental Figure 4A). In congruence with murine T cells, dysfunctional (PD-1^+^LAG-3^+^) human CD8^+^ T cells (32) expressed higher levels of lipid peroxidation– and ferroptosis activation–related genes than nondysfunctional CD8^+^ T cells in melanoma patient tumor tissues (Supplemental Figure 4, B and C). Moreover, by analyzing tumor-infiltrating CD8^+^ T cells in melanoma patients treated with PD-1 or CTLA4 checkpoint immunotherapy (36), we found that T cells from nonresponders showed higher enrichment of lipid peroxidation and ferroptosis activation genes compared with responders (Supplemental Figure 4, D and E). These findings suggest that there may be a negative correlation between IL-9 and ferroptosis and a positive correlation between ferroptosis and dysfunction in human CD8^+^ T cells.

### Tc9 cells are less susceptible to tumor- or ROS-induced lipid peroxidation and ferroptosis in the TME.

**Figure 3:**
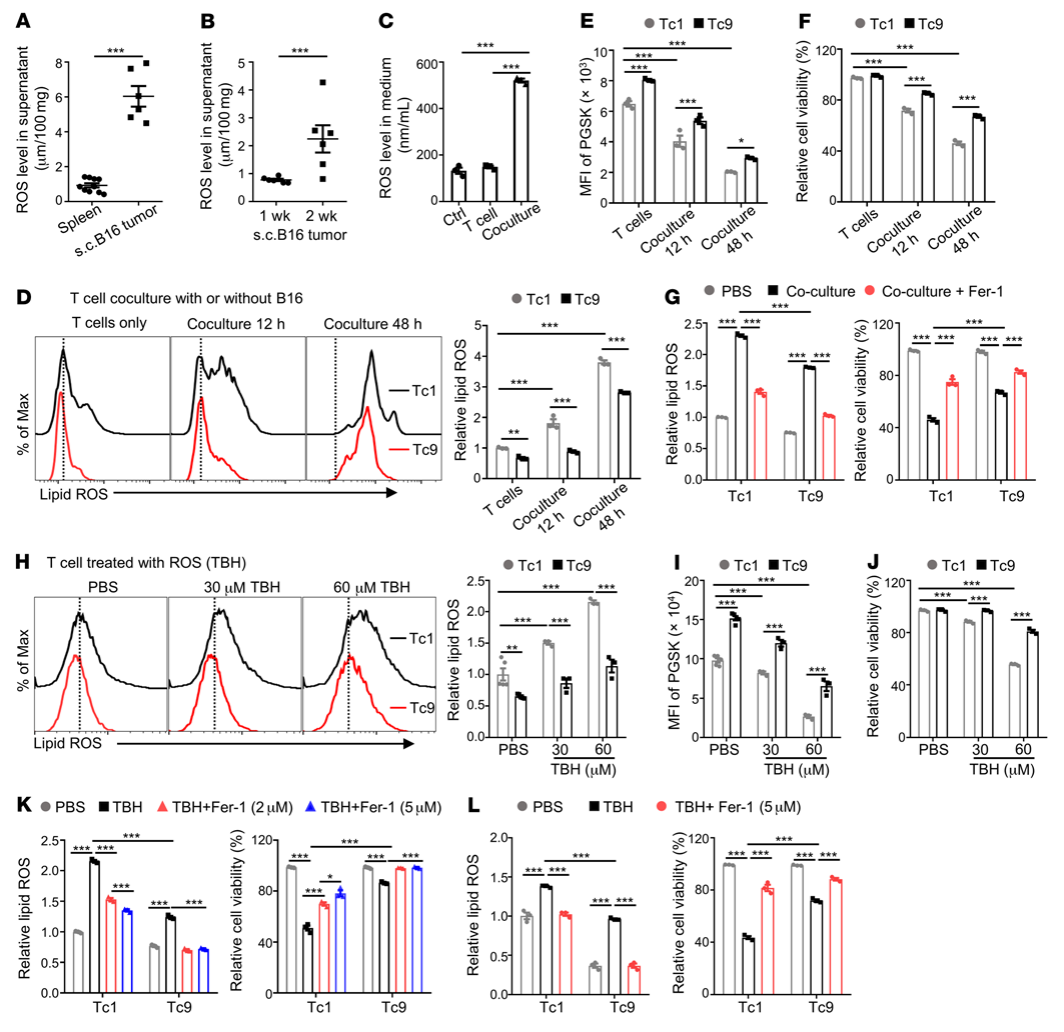
Tc9 but not Tc1 cells resist tumor- or ROS-induced lipid peroxidation and ferroptosis in the TME. (**A** and **B**) B6 mice were injected s.c. with 1 × 10^6^ B16 cells, and spleen and tumor tissues were collected on week 1 or 2 after tumor inoculation. Shown are ROS levels in the supernatants of spleens and 2-weeks-old tumors in **A** or tumors collected on week 1 or 2 in **B** (*n =* 6). (**C**–**F**) Pmel-1 CD8^+^ T cells were isolated and stimulated with anti-CD3/anti-CD28 antibodies in the presence of corresponding polarizing cytokines. B16 cells cocultured with Tc1 or Tc9 cells for indicated time on day 4 of polarization. Shown are ROS level in control medium, T cell culture medium, or T cell–B16 coculture 48-hour medium in **C**, relative lipid ROS, cellular iron level, and viability in Tc1 or Tc9 cells (*n =* 3). (**G**) B16 cells were cocultured with Tc1 or Tc9 cells for 48 hours with or without Fer-1. Relative lipid ROS and cell viability are shown (*n =* 3). (**H**–**J**) Tc1 or Tc9 cells were treated with ROS inducer TBH at indicated concentrations. Relative lipid ROS, cellular iron level, and viability after 16-hour culture are shown (*n =* 3–5). (**K**) Relative lipid ROS and cell viability were detected in Tc1 and Tc9 cells after treatment with 60 μM TBH with or without Fer-1 at indicated concentrations (*n =* 3). (**L**) Relative lipid ROS and cell viability in Tc1 and Tc9 cells after treatment with 60 μM TBH with or without DFO (*n =* 3). Data are presented as mean ± SEM. *P < 0.05; **P < 0.01; ***P < 0.001 by unpaired, 2-tailed Student’s *t* test in **A** and **B**, 1-way ANOVA followed by Dunnett’s test in **C**, and 2-way ANOVA in the other panels.

As Tc9 but not Tc1 cells display reduced ferroptosis in the TME, we wanted to identify the cause of induction of ferroptosis in T cells in the TME. Because previous studies reported that tumor cells are induced to undergo ferroptosis in vitro via ROS-mediated stress (37), we wanted to determine whether ROS also induce CD8^+^ T cell ferroptosis in the TME. First, we detected ROS levels in spleens and tumor tissues of tumor-bearing mice at different time points and showed that s.c. B16 tumors had a much higher level of ROS compared with spleens of the mice (Figure 3A). Interestingly, ROS levels were positively associated with tumor progression because their levels were significantly upregulated in 2-week tumors compared with 1-week tumors (Figure 3B). These findings indicate that tumor tissues are enriched with ROS.

Second, we cocultured CD8^+^ T cells with B16 tumor cells to simulate the TME and observed highly increased ROS in coculture media compared with T cell culture medium (Figure 3C). Tc1 cells, after coculture with B16 tumor cells in vitro, had higher lipid peroxidation (Figure 3D), iron levels, and cell death (Figure 3, E and F) than Tc9 cells, which are consistent with our in vivo findings. In addition, when we added the ferroptosis inhibitor Fer-1 into the coculture system, tumor-induced lipid peroxidation and ferroptosis were rescued in both Tc1 and Tc9 cells (Figure 3G). To confirm that ROS can directly induce CD8^+^ T cell ferroptosis, we treated Tc1 and Tc9 cells with *tert*-butyl hydroperoxide (TBH), a commonly used ROS inducer. Consistent with coculture results, TBH, in a dose-dependent manner, induced lipid peroxidation, iron accumulation, and cell death in CD8^+^ T cells. However, Tc9 cells, compared with Tc1 cells, were significantly less susceptible to TBH-induced lipid ROS production and ferroptosis (Figure 3, H–J). The addition of the ferroptosis inhibitor Fer-1 or iron chelator DFO rescued TBH-induced lipid peroxidation and ferroptosis in both Tc1 and Tc9 cells (Figure 3, K and L). These results indicate that Tc9 cells have greater resistance to tumor- or ROS-induced ferroptosis than Tc1 cells.

### Increased fatty acid oxidation in Tc9 cells contributes to reduced lipid peroxidation and ferroptosis.

**Figure 4:**
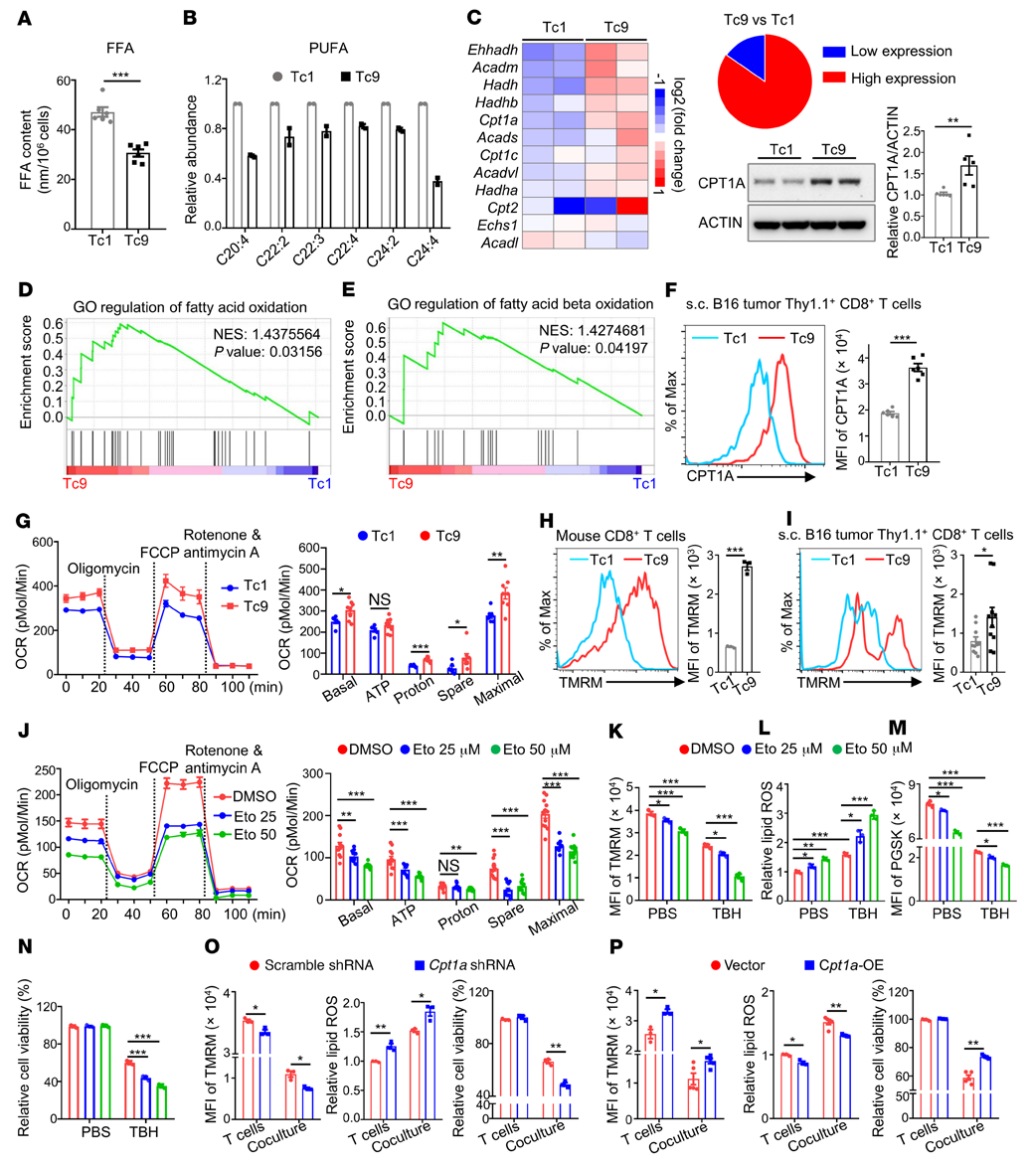
Increased fatty acid oxidation is required for reduced lipid peroxidation and ferroptosis in Tc9 cells. (**A**) Free fatty acid (FFA) contents (*n =* 6) and (**B**) polyunsaturated fatty acid (PUFA) levels (*n =* 2) in tumor-infiltrating Tc1 or Tc9 cells from s.c. B16 tumor–bearing mice. (**C**) Heatmap of fatty acid oxidation–related gene expression in tumor-infiltrating Tc1 and Tc9 cells and *Cpt1a* expression in in vitro–polarized Tc1 and Tc9 cells. (**D** and **E**) GSEA of indicated gene sets in tumor-infiltrating Tc9 cells compared with Tc1 cells. GO, Gene Ontology; NES, normalized enrichment score. (**F**) *Cpt1a* expression in Tc1 and Tc9 cells from s.c. B16 tumors (*n =* 6). (**G**) Statistical analysis of oxygen consumption rates (OCRs) in Tc1 and Tc9 cells with indicated treatments (*n =* 6–8). (**H** and **I**) TMRM intensity of in vitro–polarized (*n =* 3) or tumor-infiltrated (*n =* 10) Tc1 and Tc9 cells. (**J**) OCR of Tc9 cells treated with etomoxir (Eto) at indicated concentrations (*n =* 12). (**K**–**N**) TMRM intensity, relative lipid ROS and iron levels, and relative cell viability in Tc9 cells treated with ROS inducer TBH in combination with or without etomoxir at indicated concentrations for 16 hours. Before TBH treatment, Tc9 cells were treated with etomoxir for 12 hours (*n =* 3). (**O**) TMRM intensity, relative lipid ROS levels, and cell viability in scramble shRNA– or *Cpt1a* shRNA lentivirus–transduced Tc9 cells cocultured with B16 cells for 48 hours (*n =* 3). (**P**) TMRM intensity, relative lipid ROS levels, and cell viability in vector- or *Cpt1a*-overexpressing (*Cpt1a*-OE) transduced Tc9 cells cocultured with B16 cells for 48 hours (*n =* 3–5). Data are presented as mean ± SEM. *P < 0.05; **P < 0.01; ***P < 0.001 by 1-way ANOVA followed by Dunnett’s test in **J**–**N** and unpaired, 2-tailed Student’s *t* test in the other panels.

We sought to elucidate the mechanisms underlying reduced lipid peroxidation and ferroptosis in Tc9 cells. We reported that free fatty acids, especially arachidonic acid (AA, C20:4), induce CD8^+^ T cell ferroptosis through uptake of free fatty acids (26). To determine whether free fatty acids play a role in Tc9 cell ferroptosis, we first measured free fatty acid content in Tc1 and Tc9 cells and determined their role in T cells. Indeed, the levels of free fatty acids, especially AA, one of the polyunsaturated fatty acids (PUFAs), were significantly lower in Tc9 than in Tc1 cells (Figure 4, A and B). Additionally, both free fatty acids and AA induced more ferroptosis in Tc1 than in Tc9 cells (Supplemental Figure 5, A–C). Second, we sought to understand how Tc9 cells maintain a lower free fatty acid and AA content. As the content of cellular free fatty acids is controlled by the balance of fatty acid synthesis, uptake, and oxidation (38), we determined and compared fatty acid–related gene expression in Tc1 and Tc9 cells by RNA-Seq. We observed that expression of genes related to fatty acid oxidation, especially β-oxidation, was significantly higher in Tc9 than in Tc1 cells (Figure 4C), whereas there was no significant difference in the expression of genes regulating fatty acid synthesis or uptake between these cells (Supplemental Figure 5D). These results were confirmed by RT-PCR (Supplemental Figure 5, E and F). In line with these findings, Western blotting and flow cytometry analyses showed that the expression of carnitine palmitoyltransferase I (CPT1A), a rate-limiting enzyme of fatty acid β-oxidation, was significantly higher in Tc9 than in Tc1 cells (Figure 4C and Supplemental Figure 5G), and GSEA results showed that signaling pathways regulating fatty acid oxidation and fatty acid β-oxidation were enriched in Tc9 cells as compared with Tc1 cells (Figure 4, D and E). In addition, tumor-infiltrating Tc9 cells expressed a higher level of CPT1A than Tc1 cells (Figure 4F).

As fatty acid β-oxidation mainly exists in and fuels mitochondria, we examined mitochondrial function and activity of T cells by mitochondrial stress test and tetramethylrhodamine methyl ester (TMRM) staining. Tc9 cells had increased basal and maximal oxygen consumption rates (OCRs), spare respiratory capacity (the quantitative difference between the maximal OCR and the initial basal OCR that indicates an enhanced mitochondrial function and extra capacity available in the cells to produce energy in response to increased stress; refs. 39, 40), and mitochondrial mass compared with Tc1 cells (Figure 4G and Supplemental Figure 6A). Extracellular acidification rate (ECAR) and OCR/ECAR values were similar between Tc1 and Tc9 cells, although basal ECAR values were higher in Tc9 cells (Supplemental Figure 6, B and C). Both in vitro–polarized Tc9 cells and adoptively transferred Tc9 cells isolated from tumors and spleens displayed an enhanced mitochondrial activity compared with Tc1 cells (Figure 4, H and I, and Supplemental Figure 6, D and E). Moreover, mitochondrial activity of Tc9 cells was significantly decreased when treated with the fatty acid oxidation inhibitor etomoxir (Figure 4J). These results revealed that Tc9 cells have lower fatty acid content and increased fatty acid oxidation and mitochondrial activity than Tc1 cells.

To determine whether increased fatty acid oxidation is responsible for reduced lipid peroxidation and ferroptosis in Tc9 cells, we treated Tc9 cells with the ROS inducer TBH, with or without the CPT1A inhibitor etomoxir. We found that etomoxir, in a dose-dependent manner, significantly negated TBH’s effects in inhibiting mitochondrial activity (Figure 4K) and inducing lipid peroxidation (Figure 4L), cellular iron accumulation (Figure 4M), and ferroptosis (Figure 4N) in Tc9 cells. To exclude the potential off-target effects of etomoxir, we knocked down *Cpt1a* in Tc9 cells (Supplemental Figure 6F) and observed that *Cpt1a* deficiency also significantly diminished the TBH- or RSL3-induced (positive control) mitochondrial activity decrease and lipid peroxidation and ferroptosis in Tc9 cells (Supplemental Figure 6G). In addition, after coculture with B16 cells, *Cpt1a*-knockdown Tc9 cells were more vulnerable to tumor-induced decreases in mitochondrial activity and increases in lipid peroxidation and ferroptosis than control Tc9 cells (Figure 4O), while *Cpt1a* overexpression (Supplemental Figure 6H) effectively rescued Tc9 cells from tumor-induced decreases in mitochondrial activity and increases in lipid peroxidation and ferroptosis (Figure 4P). These results clearly indicate that increased fatty acid oxidation is responsible for reduced lipid peroxidation and ferroptosis in Tc9 cells.

### Tc9 cells resist tumor- or ROS-induced ferroptosis through the IL-9/STAT3/fatty acid oxidation pathway.

**Figure 5:**
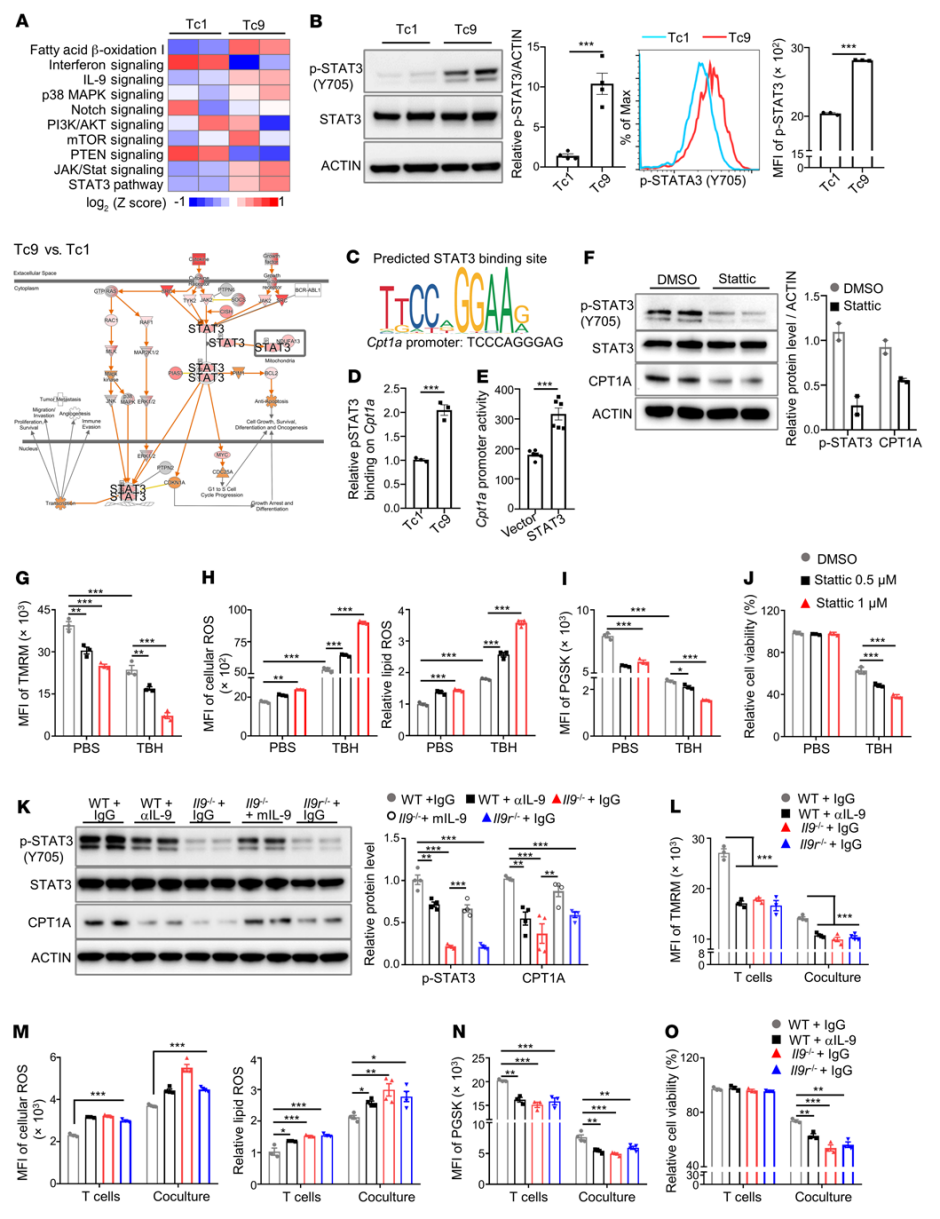
IL-9 regulates fatty acid oxidation through p-STAT3 signaling to resist tumor- or ROS-induced ferroptosis in Tc9 cells. (**A**) IPA of canonical signaling pathways (upper panel) and STAT3 pathway (low panel) in Tc9 versus Tc1 cells isolated from tumors. Red indicates upregulation. (**B**) p-STAT3 expression in polarized Tc1 and Tc9 cells (*n =* 3–4). (**C** and **D**) Predicted STAT3 binding site and *Cpt1a* promoter sequence, and relative p-STAT3 binding on the *Cpt1a* promoter in Tc1 and Tc9 cells by ChIP detection (*n =* 3). (**E**) Dual-luciferase-reporter analysis for the activation of the *Cpt1a* promoter by vector- and STAT3-overexpressing plasmid in HEK293T cells (*n =* 6). (**F**) p-STAT3 and CPT1A expression in polarized Tc9 cells treated with Stattic (1 μM) (*n =* 2). (**G**–**J**) TMRM intensity, cellular ROS level, and relative lipid ROS, iron level, and relative cell viability in polarized Tc9 cells treated with TBH with or without Stattic at indicated concentrations for 16 hours. Before TBH treatment, Tc9 cells were treated with Stattic for 4 hours (*n =* 3). (**K**) p-STAT3 and CPT1A expression in polarized Tc9 cells from Pmel-1 mice (WT) treated with IgG or anti–IL-9 (αIL-9), from Pmel-1 *Il9*-knockout mice (*Il9^–/–^*) treated with IgG or mouse recombinant IL-9, or from Pmel-1 *Il9r*-knockout mice (*Il9r^–/–^*) treated with IgG (*n =* 4). (**L**–**O**) TMRM intensity, cellular ROS level, relative lipid ROS level, iron level, and relative cell viability in polarized Tc9 cells from indicated mice cocultured with B16 cells for 48 hours (*n =* 3–4). Data are presented as mean ± SEM. *P < 0.05; **P < 0.01; ***P < 0.001 by unpaired, 2-tailed Student’s *t* test in **B**, **D**, and **E** and 1-way ANOVA followed by Dunnett’s test in the other panels.

To determine the molecular mechanisms underlying the upregulated fatty acid oxidation in Tc9 cells, we performed RNA-Seq and Ingenuity Pathway Analysis (IPA) of Tc9 and Tc1 cells. Consistently, the fatty acid β-oxidation pathway was activated in Tc9 cells (Figure 5A). Importantly, we found that the JAK/STAT signaling pathways, especially the STAT3 pathway, were significantly activated in Tc9 cells compared with Tc1 cells (Figure 5A and Supplemental Figure 7A). To confirm these results, we examined the expression of phosphorylated STAT3 (p-STAT3) by Western blotting and flow cytometry and showed that p-STAT3 levels were significantly increased in Tc9 cells compared with Tc1 cells (Figure 5B). As a previous study showed that STAT3 can activate CPT1B and CPT1B is mainly expressed in heart and skeletal muscle cells (41), we determined whether p-STAT3 could activate CPT1A in Tc9 cells. First, we analyzed the *Cpt1a* promoter and identified a potential binding site for p-STAT3. To determine whether binding of p-STAT3 to the *Cpt1a* promoter is increased in Tc9 cells, a chromatin immunoprecipitation (ChIP) assay was performed and showed that p-STAT3 had increased binding to the *Cpt1a* promoter in Tc9 but not in Tc1 cells (Figure 5, C and D). Second, a luciferase reporter assay showed that STAT3 activated *Cpt1a* gene transcription (Figure 5E). Third, when we treated Tc9 cells with Stattic, a p-STAT3 inhibitor, CPT1A expression was decreased along with an inactivation of p-STAT3 (Figure 5F and Supplemental Figure 7B). In line with this result, CPT1A expression was increased after treating Tc9 cells with the p-STAT3 inducer IL-6 (Supplemental Figure 7C). Moreover, when we treated Tc9 cells with the ROS inducer TBH in the presence of Stattic, Tc9 cell mitochondrial activity was decreased (Figure 5G) and levels of cellular ROS, lipid peroxidation (Figure 5H), cellular iron (Figure 5I), and ferroptosis (Figure 5J) were increased. Altogether, these results indicate that STAT3 signaling induces CPT1A expression, which in turn protects Tc9 cells from ROS- or tumor-induced ferroptosis.

Next, we attempted to identify upstream factors of STAT3 signaling that activate CPT1A expression. As (a) the expression of common inducers of STAT3 (*Il6*, *Il10*, and *Il27*) was comparable between Tc1 and Tc9 cells, (b) IL-9 is the most featured cytokine in Tc9 cells, and (c) IPA analysis showed that there was a strong crosstalk between IL-9 signaling and the STAT3 pathway in Tc9 cells (Supplemental Figure 7, D and E), we investigated whether IL-9 could activate STAT3 signaling. We examined p-STAT3 expression in Tc9 cells with or without IL-9 and showed that p-STAT3 and CPT1A levels were dramatically decreased when Tc9 cells were treated with IL-9–neutralizing antibody or Tc9 cells were derived from *Il9*-knockout (*Il9^–/–^*) or *Il9* receptor–knockout (*Il9r^–/–^*) mice. Moreover, p-STAT3 and CPT1A expression levels were rescued in *Il9^–/–^* Tc9 cells with the addition of recombinant IL-9 (Figure 5K). To validate that the low levels of free fatty acids and PUFAs in Tc9 cells are dependent on IL-9–mediated β-oxidation, we detected the free fatty acid content in the cells by LC-MS analysis. Naive CD8^+^ T cells isolated from WT and *Il9^–/–^* mice had comparable levels of free fatty acids (including saturated, monounsaturated, and polyunsaturated) and CPT1A before polarization (Supplemental Figure 8, A and B). However, polarized WT Tc9 cells had lower levels of free fatty acids, especially PUFAs, compared with WT Tc1 cells and *Il9^–/–^* Tc9 cells (Supplemental Figure 8C). To further confirm the importance of the IL-9/IL-9R axis in maintaining low levels of lipid peroxidation and ferroptosis in Tc9 cells, we cocultured Pmel-1 WT, *Il9^–/–^*, or *Il9r^–/–^* mouse–derived Tc9 cells with B16 tumor cells and observed that mitochondrial activity was lower (Figure 5L), while cellular ROS, lipid peroxidation (Figure 5M), cellular iron levels (Figure 5N), and ferroptosis (Figure 5O) were higher in Tc9 cells from *Il9^–/–^* and *Il9r^–/–^* mice compared with T cells from WT mice. Similar results were obtained in IL-9–neutralizing antibody–treated Tc9 cells (Figure 5, L–O). Additionally, when Tc9 cells were treated with the ROS inducer TBH, *Il9^–/–^* and *Il9r^–/–^* Tc9 cells had even lower mitochondrial activity and higher levels of cellular ROS, lipid peroxidation, cellular iron, and ferroptosis (Supplemental Figure 8, D–G) than WT Tc9 cells. Specifically, when we overexpressed *Cpt1a* in *Il9^–/–^* Tc9 cells and treated them with TBH, *Cpt1a* overexpression significantly inhibited TBH-induced suppression of mitochondrial activity, production of cellular ROS, and induction of lipid peroxidation and cell death (Supplemental Figure 8, H–J). Similar results were found in Tc9 cells cocultured with B16 tumor cells (Supplemental Figure 8, K–M). Thus, these results indicate that IL-9 activates STAT3 signaling in Tc9 cells to upregulate fatty acid oxidation, leading to Tc9 cells with reduced lipid peroxidation and resistance to the induction of ferroptosis in the TME.

### Inhibiting the IL-9/STAT3/fatty acid oxidation pathway impairs longevity and antitumor ability of Tc9 cells.

**Figure 6:**
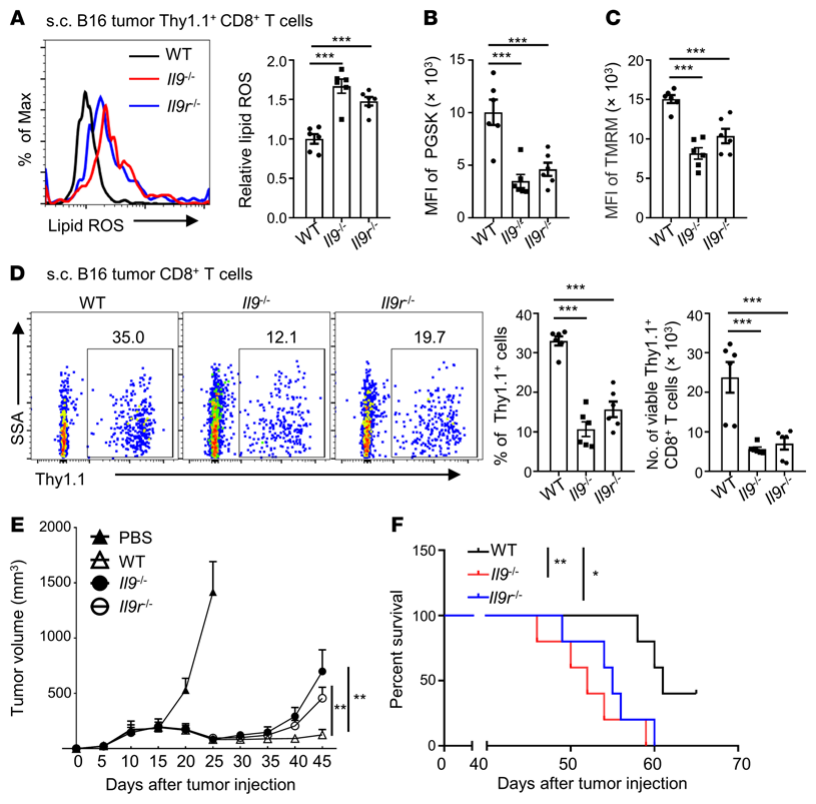
IL-9 signaling deficiency impairs the longevity and antitumor effects of Tc9 cells. Thy1.1^+^ Pmel-1 Tc9 cells from WT mice, *Il9^–/–^* mice, and *Il9r^–/–^* mice were injected into B16 tumor–bearing Thy1.2^+^ B6 mice with adjuvant treatments. (**A**) Relative lipid ROS, (**B**) iron level, (**C**) TMRM intensity, and (**D**) Thy1.1^+^ percentages in CD8^+^ T cells and Thy1.1^+^ CD8^+^ T cell numbers in tumors on day 45 after tumor injection (*n =* 6, two pooled independent experiments). (**E** and **F**) Tumor growth and survival curve of treated mice (*n =* 5). Data are presented as mean ± SEM. Eto, etomoxir; OE, overexpressing. *P < 0.05; **P < 0.01; ***P < 0.001 by log-rank (Mantel-Cox) test in **F**, 2-way ANOVA in **E**, and 1-way ANOVA followed by Dunnett’s test in the other panels.

Because the IL-9/IL-9R axis is important for reducing lipid peroxidation and ferroptosis in Tc9 cells in vitro, we investigated whether IL-9 or IL-9R deficiency could affect the Tc9 cells’ ability to persist and control tumor growth in vivo. We adoptively transferred Tc9 cells from Thy1.1^+^ Pmel-1 WT, *Il9^–/–^*, or *Il9r^–/–^* mice into Thy1.2^+^ mice bearing s.c. B16 tumors. As expected, tumor-infiltrating *Il9^–/–^* or *Il9r^–/–^* Tc9 cells had higher lipid peroxidation (Figure 6A) and cellular iron levels (Figure 6B), and lower mitochondrial activity (Figure 6C) than WT Tc9 cells, indicating that IL-9– or IL-9 signaling–deficient Tc9 cells undergo enhanced ferroptosis in the TME. Consequently, the persistence of *Il9^–/–^* or *Il9r^–/–^* Tc9 cells in tumors (Figure 6D) or blood (Supplemental Figure 9, A–D) of treated mice was significantly impaired, and their antitumor efficacy (Figure 6, E and F) was significantly compromised in comparison with WT Tc9 cells. These results clearly demonstrate that IL-9 or IL-9R deficiency dampens the antitumor effects of Tc9 cells.

**Figure 7:**
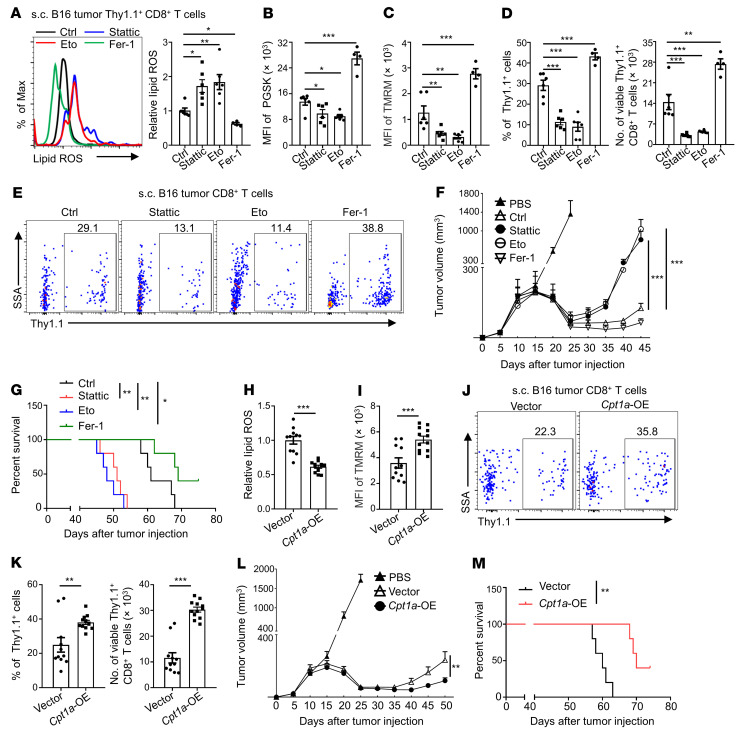
Inhibiting the STAT3/fatty acid oxidation pathway in Tc9 cells impairs their longevity and antitumor effects. (**A**–**G**) Thy1.1^+^ Pmel-1 Tc9 cells from WT mice were treated with or without Stattic (0.5 μM), etomoxir (Eto, 50 μM), or Fer-1 (5 μM) and injected into B16 tumor–bearing Thy1.2^+^ B6 mice with adjuvant treatments. Relative lipid ROS, iron level, TMRM intensity, Thy1.1^+^ percentages in CD8^+^ T cells, Thy1.1^+^CD8^+^ T cell numbers in tumors on day 45 after tumor injection (*n =* 4–6, two pooled independent experiments), and tumor growth and survival curve of treated mice (*n =* 5). (**H**–**M**) Thy1.1^+^ Pmel-1 Tc9 cells from WT mice transduced with vector or *Cpt1a* were injected into B16 tumor–bearing Thy1.2^+^ B6 mice with adjuvant treatments. Relative lipid ROS, TMRM intensity, Thy1.1^+^ percentages in CD8^+^ T cells, and Thy1.1^+^ CD8^+^ T cell numbers in tumors on day 45 after tumor injection (*n =* 11–12, two pooled independent experiments), tumor growth, and survival curve of treated mice (*n =* 5). Data are presented as mean ± SEM. Eto, etomoxir; OE, overexpressing. *P < 0.05; **P < 0.01; ***P < 0.001 by 1-way ANOVA followed by Dunnett’s test in **A**–**D**, 2-way ANOVA in **F** and **L**, log-rank (Mantel-Cox) test in **G** and **M**, and unpaired, 2-tailed Student’s *t* test in the other panels.

Finally, we determined whether inhibiting STAT3 signaling or fatty acid oxidation would affect Tc9 cell induction of ferroptosis and persistence in vivo. We injected B16 tumor–bearing mice with control, Stattic-treated (STAT3 inhibitor), etomoxir-treated (CPT1A inhibitor), or Fer-1–treated (ferroptosis inhibitor) Tc9 cells. Consistent with our in vitro results, Stattic- or etomoxir-treated tumor-infiltrating Tc9 cells had higher lipid peroxidation (Figure 7A) and iron levels (Figure 7B), lower mitochondrial activity (Figure 7C), and impaired persistence (Figure 7, D and E) than control Tc9 cells, whereas Fer-1–treated tumor-infiltrating Tc9 cells had lower lipid peroxidation (Figure 7A) and iron levels (Figure 7B), higher mitochondrial activity (Figure 7C), and enhanced persistence (Figure 7, D and E) than control Tc9 cells. Similar results were found in peripheral blood of these mice (Supplemental Figure 10, A–E). Consequently, Stattic- or etomoxir-treated Tc9 cells displayed compromised antitumor ability compared with control Tc9 cells and Fer-1–treated Tc9 cells exerted stronger antitumor ability than control Tc9 cells (Figure 7, F and G). Moreover, we constructed *Cpt1a*-overexpressing Tc9 cells and these T cells displayed further reduced lipid peroxidation (Figure 7H), enhanced mitochondrial activity (Figure 7I) and persistence (Figure 7, J and K), and exerted stronger antitumor ability than vector-control Tc9 cells (Figure 7, L and M). Similar results were found in circulating Tc9 cells (Supplemental Figure 10, F–H). Additionally, *Cpt1a*-overexpressing Tc1 cells showed reduced lipid peroxidation, enhanced mitochondrial activity, and better persistence and antitumor ability in vivo compared with vector-control Tc1 cells (Supplemental Figure 10, I–L). However, *Cpt1a-*overexpressing Tc1 cells still expressed higher levels of exhaustion markers such as PD-1 and LAG-3 compared with Tc9 cells (Supplemental Figure 10M), which may explain why *Cpt1a*-overexpressing Tc1 cells remained inferior in their antitumor ability in vivo compared with Tc9 cells.

Taken together, these results show that inhibiting the IL-9/STAT3 pathway or fatty acid oxidation worsens lipid peroxidation and ferroptosis of Tc9 cells, resulting in impaired longevity and antitumor ability in vivo, whereas inhibiting ferroptosis or enhancing fatty acid oxidation extends Tc9 cells’ persistence and antitumor effect.

## Discussion

As one of the recently described CD8^+^ T cell subsets, Tc9 cells are generated by differentiating naive CD8^+^ T cells under Th9-polarizing medium and featured by IL-9 secretion (17, 42). Tc9 cells have been detected in atopic dermatitis lesions of mice and humans (42), in the small intestine (43), and in the peripheral blood of patients with allergic asthma (44). Although IL-9–secreting Tc9 cells are less cytolytic in vitro, they produce comparable levels of IFN-γ and granzyme B as Tc1 cells in spleen and tumors after adoptive transfer. More importantly, Tc9 cells persist longer in vivo to exert superior antitumor ability than Tc1 cells (5, 17). However, why Tc9 cells persist longer than Tc1 cells in vivo remains unclear. In the present study, we uncovered what we believe is a novel mechanism for Tc9 cell persistence and survival. We found that adoptively transferred Tc9 cells contained significantly lower levels of lipid peroxidation than Tc1 cells in tumors, which was strongly correlated with the persistence of Tc9 cells. Both murine and human Tc9 cells were less susceptible to induction of ferroptosis in the TME, which is required for their longevity. Our mechanistic results revealed that IL-9 upregulates fatty acid oxidation through the STAT3 pathway to resist ROS-induced lipid peroxidation and ferroptosis in Tc9 cells. IL-9 or IL-9 signaling deficiency or inhibiting STAT3/fatty acid oxidation increased ferroptosis, reduced longevity, and impaired the antitumor ability of Tc9 cells. Conversely, inhibiting ferroptosis or enhancing fatty acid oxidation increased the longevity and antitumor ability of Tc9 cells. Interestingly, human tumor-infiltrating CD8^+^ T cells expressed lower IL-9 and higher lipid peroxidation– and ferroptosis-related genes than circulating CD8^+^ T cells in melanoma patients, which may support our conclusion.

Overwhelmingly, lipid peroxidation products are characteristic features of ferroptosis. Ferroptosis is involved in various disease conditions, especially degenerative disorders and cancer (27, 29). Induction of ferroptosis in cancer cells leads to tumor regression (27, 45) and CD8^+^ T cells can sensitize tumor cells to ferroptosis via secreted IFN-γ (46). However, little is known about ferroptosis in CD8^+^ T cells in tumor immunotherapy. We recently reported that CD36 mediates ferroptosis in CD8^+^ T cells by facilitating T cell uptake of fatty acids, which dampens intratumoral CD8^+^ T cell effector function and antitumor ability (26), and another group also observed that CD8^+^ T cells were more sensitive to ferroptosis induction than B16 and MC38 cancer cells (47). In this study, we revealed one of the mechanisms attributed to the increased persistence and antitumor effects of Tc9 cells in vivo, when compared with Tc1 cells: Tc9 cells are more resistant to tumor- or TME-induced lipid peroxidation and ferroptosis. Our findings also shed light on the underlying mechanism of why the in vivo lifespan of Tc1 cells is short after adoptive transfer.

We elucidated the mechanisms of CD8^+^ T cell ferroptosis in the TME. Previous studies showed that deficiency of glutamine or cysteine induces tumor cell ferroptosis (45); however, these factors did not have any effects on CD8^+^ T cell ferroptosis. Elevated levels of ROS have been reported in the TME (25) and ROS induces ferroptosis in p53-deficient cells in vitro (37). Consistent with these results, we found that ROS levels were elevated in s.c. B16 tumors and CD8^+^ T cell–B16 coculture media, which led to induction of ferroptosis in Tc1, but less so in Tc9 cells.

AA and adrenic acid–esterified phosphatidylethanolamines have been reported to be the substrates for lipid peroxidation (48, 49). We recently found that fatty acids, especially AA, induce CD8^+^ T cell lipid peroxidation and ferroptosis in the TME (26). Lipid peroxidation occurs when oxidants attack lipids containing carbon-carbon double bond(s), especially PUFAs (28). Cellular PUFAs are mainly converted from dietary fatty acids and increased fatty acid oxidation decreases fatty acid content, including PUFAs (50, 51). Prior work in tumor cells has demonstrated that impaired fatty acid oxidation leads to accumulation of damaged lipids and induction of ferroptosis (48, 52). Furthermore, knocking down 2,4-dienoyl–CoA reductase (DECR1), an enzyme involved in fatty acid oxidation, causes cellular accumulation of PUFAs and enhances mitochondrial oxidative stress and lipid peroxidation in human prostate cancer (53). In this study, we discovered that Tc9 cells have higher fatty acid oxidation and mitochondrial activity and lower PUFA levels than Tc1 cells, which may explain why Tc9 cells are endowed with lower lipid peroxidation and resistance to ROS-induced ferroptosis. As CPT1A is a rate-limiting enzyme of fatty acid oxidation that mainly occurs in mitochondria (40), inhibiting fatty acid oxidation also decreases mitochondrial activity and respiratory capacity. Importantly, inhibiting fatty acid oxidation in Tc9 cells impaired, while overexpressing *Cpt1a* in Tc9 cells enhanced, their longevity and antitumor ability in vivo. In nutrient-deprived TMEs, metabolic fitness of CD8^+^ T cells is critical to their persistence and antitumor efficiency (24, 54). Tumor-infiltrating effector CD8^+^ T cells engage glycolysis and glutaminolysis to sustain their demand for proliferation and effector function, but cancer cells also have a high demand for glycolysis and glutaminolysis, and thus nutrient competition leads to the insufficiency of glucose and glutamine in the TME (54, 55). Consequently, effector T cells become exhausted or dead. Different from effector T cells, memory CD8^+^ T cells have lower glycolytic rates and higher reliance on fatty acid oxidation to generate ATP, and this metabolic flexibility facilitates their survival in the TME but limits their secretion of cytotoxic molecules (55). As for Tc9 cells, they share some metabolic features of memory T cells such as enhanced fatty acid oxidation and mitochondrial activity and may sustain comparable glycolysis to that of Tc1 cells. This metabolic fitness of Tc9 cells confers them with great antitumor ability.

Recent studies reported that fatty acid oxidation is required for CD8^+^ memory T cell maintenance (39, 40) and STAT3 is important for memory T cell development (56, 57). These observations indicate that there may be a link between STAT3 signaling and fatty acid oxidation. Here, we showed that p-STAT3 activated fatty acid oxidation in Tc9 cells by direct binding to the *Cpt1a* promoter. STAT3 is a transcription factor and plays a critical yet controversial role in regulating T cell function. Some studies showed that STAT3 promotes T cell survival and STAT3 mutation results in a decrease in memory CD8^+^ T cell subsets in vivo (56, 57). Others reported that STAT3 dampens cytotoxic cytokine expression in Tc17 cells (58). In this study, we observed that activating p-STAT3 in response to IL-9 upregulates fatty acid oxidation in Tc9 cells and confers Tc9 cell resistance to ROS-induced ferroptosis. Conversely, inhibiting p-STAT3 impairs the longevity and antitumor ability of Tc9 cells.

To conclude, this study identifies a mechanism underlying the persistence and antitumor activity of Tc9 cells in the TME. Enhanced fatty acid oxidation through STAT3 signaling activation protects Tc9 cells from tumor- or TME-induced lipid peroxidation and ferroptosis. Our study suggests that inhibiting ferroptosis or enhancing fatty acid oxidation may be an effective strategy to improve the antitumor efficiency of ACT-based immunotherapy. Our study also highlights the importance of targeting lipid metabolism or lipid peroxidation in T cells to improve their clinical effectiveness in cancer immunotherapy.

## Methods

### Contact for reagent and resource sharing.

Further information and requests for resources and reagents should be directed to and will be fulfilled by the lead contact, Qing Yi (QYi@houstonmethodist.org).

### Reagents and plasmids.

Antibodies for flow cytometry against mouse and human CD8 (clone 53-6.7 or SK1), DAPI, PI, Thy1.1 (clone OX-7), CD279 (PD-1, clone RMP1-30), CD223 (LAG-3, clone C9B7W) were purchased from BioLegend. Mouse anti-CPT1A antibodies were purchased from Abcam (catalog ab128568), and antibodies against p-STAT3 (catalog 9145) and STAT3 (catalog 4904) were purchased from Cell Signaling Technology. Cytokines (including recombinant human IL-2, mouse and human IL-4, human TGF-β, mouse IL-6) were purchased from R&D Systems. Neutralizing antibodies against mouse IFN-γ (catalog BP0055), IL-9 (catalog BE0181), or human IFN-γ (catalog BE0235) were purchased from Bio X Cell. Ferrostatin-1, 1*S*,3*R*-RSL3, FIN56, DFO, and Free Fatty Acid Quantitation Kit were purchased from Sigma-Aldrich. Lipid Peroxidation Assay Kit including BODIPY 581/591 C11 (D3861) and BODIPY 665/676 (B3932), Phen Green SK (PGSK, P14313), CM-H_2_DCFDA (C6827), DMEM medium, fetal bovine serum, penicillin-streptomycin, L-glutamine, 2-mercaptoethanol, CountBright Absolute Counting Beads (C36950), and Dynabeads Human T-Activator CD3/CD28 for T Cell Expansion and Activation (11131D) were purchased from Thermo Fisher Scientific. SimpleChIP Plus Enzymatic Chromatin IP kit (9005) was purchased from Cell Signaling Technology. Dual-Luciferase Reporter Assay System was purchased from Promega. EasySep Mouse Naive CD8^+^ T Cell Isolation Kit (catalog 19858) and EasySep Human Naive CD8^+^ T Cell Isolation Kit (catalog 17968) were purchased from Stemcell Technologies. Hgp100_25–33_ peptide was purchased from Genscript. DCF ROS/RNS Assay Kit (ab238535) and cellular reactive oxygen species detection assay kit (ab186029) were purchased from Abcam. All assays were conducted according to the manufacturers’ protocol unless otherwise indicated.

### Mice.

C57BL/6 and B6.Cg-Thy1a/Cy Tg(TcraTcrb)8Rest/J (Pmel-1) mice were purchased from The Jackson Laboratory. *Il9^–/–^* mice on the B6 background were provided by Dong Chen from Tsinghua University in Beijing, China. *Il9r^–/–^* mice on the B6 background were generated as described previously (59). Pmel-1 *Il9^–/–^* mice and Pmel-1 *Il9r^–/–^* mice were produced by crossing Pmel-1 mice with *Il9^–/–^* mice or *Il9r^–/–^* mice.

### Cell purification and culture.

Murine B16 and MC38 cell lines were purchased from ATCC. MC38 cells transduced with Hgp100 (MC38-gp100) were cultured in DMEM supplemented with 10% heat-inactivated fetal bovine serum, 100 U/mL penicillin-streptomycin, and 2 mM L-glutamine. Naive CD8^+^ T cells were isolated using the EasySep Mouse Naive CD8^+^ T Cell Isolation Kit. Cells were primed with plate-bound anti-CD3 (clone 17A2, 2 μg/mL) and soluble anti-CD28 (clone 37.51, 1 μg/mL) antibodies under Tc1- or Tc9-polarizing conditions. After 3 days of polarization, cells were transferred to new wells and cultured in standard T cell medium (RPMI 1640 medium supplemented with 10% heat-inactivated fetal bovine serum, 100 U/mL penicillin-streptomycin, 2 mM L-glutamine, and 50 μM 2-mercaptoethanol) with rhIL-2 (10 ng/mL) for another 2 days. In some experiments, splenocytes from Pmel-1 mice were directly stimulated with Hgp100_25–33_ peptide in corresponding polarizing medium. Tc9 cells were polarized in T cell medium supplemented with IL-4 (10 ng/mL), TGF-β (1 ng/mL), and anti–IFN-γ monoclonal antibody (clone XMG1.2, 20 μg/mL), while Tc1 cells were polarized in T cell medium supplemented with 10 ng/mL recombinant human IL-2 (rhIL-2).

For Fer-1 or DFO treatment, reagents were added to medium 1 hour before indicated treatments on day 4 and cells were cultured for another 16 to 48 hours. For RSL3, FIN56, and TBH treatment, reagents were added to culture medium on day 4 and cells were cultured for another 16 to 24 hours. For adoptive transfer, Stattic (0.5 μM), etomoxir (50 μM), or Fer-1 (5 μM) was added to medium 48 hours before transfer.

### Human CD8^+^ T cell isolation and culture.

Buffy coats of healthy donors were purchased from the Gulf Coast Regional Blood Center. Written informed consent was obtained for all subjects. PBMCs were isolated from blood by density gradient cell separation. Naive CD8^+^ T cells were isolated from PBMCs by using an EasySep Human Naive CD8^+^ T Cell Isolation Kit and stimulated with human T-activator CD3/CD28 Dynabeads (25 μL/million cells) under Tc1- or Tc9-polarizing conditions for 7 days before treatment. Tc9 cells were polarized in T cell medium supplemented with human IL-4 (10 ng/mL), human TGF-β (1 ng/mL), and human anti–IFN-γ antibodies (clone B133.5, 10 μg/mL), while Tc1 cells were polarized in T cell medium supplemented with 10 ng/mL rhIL-2.

### RNA-Seq.

Tumor-infiltrating Tc1 and Tc9 cells of 6 mice were sorted by flow cytometry on day 14 after injection and total RNA was extracted with the RNeasy Mini Kit and pooled into 2 samples per group. RNA samples were sent to BGI Americas for quality evaluation using an Agilent Bioanalyzer. GSEA was run for each cell subsets in preranked list mode with 1,000 permutations. The gene sets used in this study included GO_REGULATION_OF_LIPID_CATABOLIC_PROCESS, GO_NEGATIVE_REGULATION_OF_RESPONSE_TO_REACTIVE_OXYGEN_SPECIES, GO_REGULATION_OF_FATTY_ACID_OXIDATION, and GO_REGULATION_OF_FATTY_ACID_BETA_OXIDATION, which were downloaded from the GSEA website (https://www.gsea-msigdb.org/gsea/index.jsp). Lipid peroxidation and ferroptosis activation gene sets were selected from existing publications (29, 33) and are listed in Supplemental Table 1. The T cell exhaustion–associated signature gene sets (down and up) from the Broad Institute Molecular Signature Database (GSE24081_CONTROLLER_VS_PROGRESSOR_HIV_SPECIFIC_CD8_TCELL_DN and GSE24081_CONTROLLER_VS_PROGRESSOR_HIV_SPECIFIC_CD8_TCELL_UP) were used (60).

### Single-cell RNA-Seq analysis.

Single-cell RNA-Seq data were obtained from Moshe et al. (36) and Li et al. (32). To identify related gene expression and enriched pathways in CD8^+^ T cells, single-cell RNA-Seq data from peripheral blood and tumors of melanoma patients were downloaded from GSE123139. Mean gene expression was calculated as fragments per million mapped fragments (FPM) from the count matrix and then each sample was log_2_ transformed. Cells whose expression of CD8A or CD8B was greater than 0 were defined as CD8^+^ T cells. The FPM value of related genes in CD8^+^ T cells from each sample was calculated. To identify dysfunctional and nondysfunctional CD8^+^ T cells in tumors, CD8^+^ T cells in dysfunctional clusters (32) were selected as the dysfunctional group, and CD8^+^ T cells in cytotoxic and memory clusters were selected as the nondysfunctional group. For gene expression in CD8^+^ T cells from checkpoint blocker–treated melanoma patients, tumor single-cell RNA-Seq data were downloaded from GSE120575, and mean FPM values of related genes in CD8^+^ T cells from nonresponders and responders were calculated. A *t* test was used to examine gene expression differences. GSEA was used to calculate pathway enrichment.

### TCGA data analysis.

TCGA data were used to test the correlation between selected genes and patient survival. RNaseq v2mRNA expression data and clinical parameters were retrieved from TCGA (https://www.cancer.gov/tcga) using University of California, Santa Cruz Xena (https://xenabrowser.net/datapages/). Patients were stratified into 2 groups (high or low *IL9* expression) using the median of the *z*-score average (34). Patients were also stratified to high CD8^+^ cell infiltrates by *CD8A* expression (35, 61).

### RT-PCR.

Total RNA from T cells was extracted with the RNeasy Mini Kit and reverted to cDNA with the High-Capacity cDNA Reverse Transcription kit. RT-PCR was conducted with SYBR Select Master Mix (62). Expression was normalized to the expression of the mouse housekeeping gene encoding β-actin (*Actb*). The primers were from PrimerBank (https://pga.mgh.harvard.edu/primerbank/).

### Western blot assays.

Cell lysates and immunoblotting were performed as previously described (63). The primary antibodies used were anti-CPT1A from Abcam and anti-STAT3 and –p-STAT3 from Cell Signaling Technology.

### Viral transduction.

For C*pt1a* knockdown, *Cpt1a*-specific shRNA (GTTCTTCGTGACGTTAGAT) was synthesized by Sigma-Aldrich and subcloned into the pLKO.1-GFP lentiviral vector. Viruses were packaged in HEK293T cells transfected with Lipofectamine 2000 (Thermo Fisher Scientific). Viral supernatant was harvested from day 1 to day 3 after transfection, filtered with a 0.45-μm filter, concentrated with PEG-it Virus Precipitation Solution (System Biosciences), and stored at –80°C until use. Tc9 cells were mixed with virus and 10 μg/mL protamine sulfate (Sigma-Aldrich) in a 24-well plate after 18 to 24 hours of priming, followed by centrifugation at 450*g* and 32°C for 2 hours. After incubation, the medium was replaced. Three days later, GFP^+^ cells were sorted with a flow cytometer and cultured for further analysis. For C*pt1a* overexpression, cDNA encoding *Cpt1a* was purchased from OriGene and subcloned into the MigR1 retroviral vector (64). Viruses were packaged in HEK293T cells transfected with Lipofectamine 2000. Viral supernatant was harvested 48 hours after transfection and filtered. Viruses were then spinoculated at 2,000*g* for 2 hours at 32°C onto plates coated with retronectin (65). Tc9 cells were primed for 18 to 24 hours before transduction. Three days after retroviral transduction, GFP^+^ cells were sorted and cocultured with B16 cells or used for adoptive transfer experiments.

### Lipid peroxidation and viability measurements.

Experiments were performed according to the manufacturer’s protocol. Briefly, cells were incubated in a humidified chamber at 37°C with 5% CO_2_ for 30 minutes with lipid peroxidation sensor in cell culture medium. After incubation, cells were washed and examined by flow cytometry within 2 hours after staining. For BODIPY 581/591 C11 detection, the signals from both reduced C11 (PE channel) and oxidized C11 (FITC channel) were monitored. Relative lipid ROS is expressed as the ratio of oxidized to reduced BODIPY-C11 median fluorescence intensity (MFI) in cells and the data were normalized to control samples. For virus-infected GFP^+^ cells, BODIPY 665/676 was used for lipid peroxidation detection, and the shift in MFI in the oxidized (676 nm) channel was analyzed and the data were normalized to control samples (66). For cell viability assays, cells were stained with specific antibodies, resuspended in FACS buffer containing 1 μg/mL DAPI or PI for 5 minutes, and examined for DAPI- or PI-positive cells in a flow cytometer.

### ROS measurement.

For cellular ROS detection, cells were incubated in a humidified chamber at 37°C with 5% CO_2_ for 30 minutes with CM-H_2_DCFDA or Cellular Reactive Oxygen Species Detection Assay Kit (Deep Red) (Abcam, ab186029) in physiological buffer according to the manufacturer’s protocol. After incubation, cells were resuspended in tubes and examined by flow cytometry within 2 hours, and the signals from the FITC or APC channel were monitored.

For ROS levels in medium or tumor supernatants, a DCF ROS/RNS Assay Kit was used according to the manufacturer’s protocol.

### Cellular iron and TMRM staining.

Cells were washed with PBS twice and stained with 10 nM Phen Green SK or TMRM for 15–30 minutes at 37°C. After staining, cells were centrifuged and resuspended in PBS. Higher Phen Green SK fluorescence intensity indicates lower iron level.

### Flow cytometry.

For surface antibody staining, antibodies were used after Fc blocking. For lipid peroxidation, ROS, and iron staining, cells were incubated with these reagents and then washed and stained with surface antibodies. Results were acquired using a BD FACSymphony A3. Data were analyzed with FlowJo v10 software (TreeStar).

### LC-MS analysis of free and esterified fatty acids.

LS-MS analysis was conducted at the Metabolomics Core at the University of Texas MD Anderson Cancer Center. Tumor-infiltrating Tc1 and Tc9 cells of 6 mice were sorted by flow cytometry on day 35 after injection and pooled into 2 samples per group. For free fatty acid detection, samples were homogenized and mixed with an internal standard mixture in ice-cold methanol. Extracted free fatty acids were converted to acyl chloride intermediates and detected as previously described (26).

### ChIP assay.

SimpleChIP Plus Enzymatic Chromatin IP kits were used for ChIP assays according to the manufacturer’s protocol. Chromatin was extracted from Tc1 and Tc9 cells after being polarized for 3 days and fixed with formaldehyde. For ChIP, anti–p-STAT3 monoclonal antibodies were used at 1:50 dilution and isotype-matched control antibodies were from Cell Signaling Technology at a 1:50 dilution. The precipitated DNA was analyzed by RT-PCR with *Cpt1a* promoter primers forward (5′-TGTTCCGCAGATGAGGGTTC-3′) and reverse (5′-TGTTGGGAAGAACGGCTTGT-3′).

### Luciferase reporter assay.

Mouse *Cpt1a* promoter (nucleotides –1,000 to 100 relative to the transcription start site) was synthesized and subcloned into the pGL4.10 vector. Luciferase activity was measured with the Dual-Luciferase Reporter Assay System according to the manufacturer’s instructions.

### Metabolism assays.

For mitochondrial stress assays, T cells were suspended in Seahorse XF medium and incubated in standard culture conditions for 60 minutes, and switched to a CO_2_-free incubator for another 30 minutes, followed by measuring mitochondrial stress under basal conditions and in response to 1.5 μM oligomycin, 1.5 μM fluoro-carbonyl cyanide phenylhydrazone (FCCP), 50 nM rotenone, and 0.5 μM rotenone/antimycin using a Seahorse XF-96 Extracellular Flux Analyzer (12).

### Tumor models.

For the s.c. B16 tumor model (Figure 1, A–I, Figure 2, A–F, J, and K, Figures 6 and 7, Supplemental Figure 1A, Supplemental Figure 3, G–K, and Supplemental Figures 9 and 10), mice (5–10/group) were injected s.c. in the right hind flank with 6 × 10^5^ B16 tumor cells. For the s.c. MC38-gp100 model (Figure 1, J and K, and Supplemental Figure 1, G–J), mice (6/group) were injected s.c. in the right hind flank with 2 × 10^6^ MC38-gp100 tumor cells. On day 9, one dose of cyclophosphamide (CTX, Sigma-Aldrich) was given intraperitoneally (i.p.) at 250 mg/kg body weight. On day 10 after tumor injection, mice were treated with intravenous (i.v.) injection of 2 × 10^6^ indicated CD8^+^ T cells, followed by i.v. injection of 5 × 10^5^ peptide-pulsed bone marrow–derived dendritic cells and 4 doses of rhIL-2 (6 × 10^5^ U), as previously described (17). Tumor size was calculated as 0.5 × length × width^2^. Tail vein blood was collected, and mice were sacrificed on indicated days and tumor tissue and spleens were collected for further analysis. For the B16 melanoma lung metastatic model (Figure 1, L and M, and Supplemental Figure 1, K–M), mice (6/group) were injected i.v. with 2 × 10^5^ B16 cells. After 12 days, tumor-bearing mice were injected i.v. with 3 × 10^6^ Tc1 or Tc9 cells. Mice were sacrificed and analyzed 5 to 7 days after T cell transfer and tumor tissue and spleens were collected for further analysis. The absolute number of transferred T cells in tumors (normalized to 100 mg tissue) was calculated by CountBright absolute counting beads or by multiplying the frequency of indicated positive cells in total cells.

### Data and materials availability.

The RNA-Seq data are available in NCBI’s Gene Expression Omnibus (GEO GSE176291).

### Statistics.

Statistical analyses were performed with GraphPad Prism, version 8.0. Statistical significance for multiple comparisons was assessed by 1-way or 2-way ANOVA followed by either Dunnett’s or Tukey’s test, while an unpaired 2-tailed Student’s *t* test was used for single comparisons and log-rank (Mantel-Cox) test was used for survival curves. *P* values less than 0.05 were considered significant.

### Study approval.

All mouse experiments complied with protocols approved by the Institutional Animal Care and Use Committee of the Houston Methodist Research Institute. The human PBMC study was approved by the Institutional Review Board of the Houston Methodist Research Institute.

## Author contributions

QY and LX initiated the study, designed the experiments, and wrote the manuscript. LX and XM performed the experiments and statistical analyses. LY performed bioinformatics analysis. PS, WX, EB, QW, MX, and JQ provided reagents and important suggestions. MY assisted in generating transgenic mice.

## Supplementary Material

Supplemental data

Supplemental table 1

## Version 1 02/22/2022

In-Press Preview

## Version 2 04/01/2022

Electronic publication
