# Supplementary material for: IL-9/STAT3/fatty acid oxidation–mediated lipid peroxidation contributes to Tc9 cell longevity and enhanced antitumor activity
Source: J Clin Invest. 2022 Apr 1;132(7):e153247. doi: 10.1172/JCI153247 (PMC8970676; doi:10.1172/JCI153247)

Supplementary Figure 1. Tc9 cells have lower ROS production and similar ROS scavenging ability compared to Tc1 cells.

(**A**) Survive condition of Tc1 or Tc9 cells treated mice (n = 7). (**B**) Polarized Tc1 and Tc9 cells were treated with TBH (100 μM) and then washed with PBS. Cellular ROS levels were detected and ROS scavenging rates [(ROS level at 0.5 hour - ROS level at 1.5 hours) / Time] are shown (n = 3). (**C**) Polarized Tc1 and Tc9 cells were treated with N-Acetyl Cysteine (NAC, 50 μM) and then washed with PBS. Cellular ROS levels were detected and ROS production rates [(ROS level at 17 hour -ROS level at 1 hour)/ Time] are shown (n = 3). Data are presented as mean ± SEM. *p < 0.05; ***p < 0.001 with log-rank (Mantel–Cox) test in **A** and unpaired 2-tailed Student's *t* test in other panels.

Supplementary Figure 2. Persistence of adoptively transferred CD8$^+$ T cells in TME is negatively correlated with their lipid peroxidation.

(**A**) Tumor-infiltrating Tc1 or Tc9 cells were divided into PD-1$^+$ and PD-1$^-$ groups and analyzed for the ratio of PD-1$^+$ and PD-1$^-$ (n = 10). (**B**) GSEA of exhaustion associated gene enrichment in tumor-infiltrating Tc1 or Tc9 cells. (**C-F**) Thy1.1$^+$ Pmel-1 Tc1 or Tc9 cells were i.v. injected into s.c. B16 tumor-bearing Thy1.2$^+$ B6 mice with adjuvant treatments. Thy1.1$^+$ percentages in CD8$^+$ T cell and relative lipid ROS of Tc1 and Tc9 cells in peripheral blood and spleens (n = 8-10). (**G-J**) Thy1.1$^+$ Pmel-1 Tc1 or Tc9 cells were i.v. injected into MC38-gp100 tumor-bearing Thy1.2$^+$ B6 mice with adjuvant treatments. Tumor growth curve, Thy1.1$^+$ percentages in CD8$^+$ T cell, and relative lipid ROS of Tc1 and Tc9 in spleens (n = 6). (**K-M**) Thy1.1$^+$ Pmel-1 Tc1 or Tc9 cells were i.v. injected into Thy1.2$^+$ B6 mice bearing lung B16 tumors. Tumor foci in lung, Thy1.1$^+$ CD8$^+$ T cell percentages, and relative lipid ROS of Tc1 and Tc9 in spleens (n = 6). Data are presented as mean ± SEM. *p < 0.05; **p < 0.01; ***p < 0.001 with unpaired 2-tailed Student's *t* test.

Supplementary Figure 3. Reduced lipid peroxidation and ferroptosis are required for the persistence of Tc1 and Tc9 cells in TME.

(**A-C**) Mouse $CD8^+$ T cells were isolated from Pmel-1 $Thy1.1^+$ B6 mice and polarized with $Hgp100_{25-33}$ peptide under corresponding conditions. Relative *Trf* and *Tfrc* mRNA expression, relative lipid ROS and iron level in Tc1 and Tc9 cells on day 4 of polarization. (**D-E**) Polarized mouse Tc1 and Tc9 cells were cultured with IL-2 for another 5 days and then re-stimulated with CD3/CD28 antibodies. Relative lipid ROS and cell death are shown. (**F**) Relative cell viability in polarized mouse Tc1 and Tc9 cells treated with Fin56 under indicated concentrations. (**G-H**) Relative lipid ROS and $Thy1.1^+$ percentages in $CD8^+$ T cell from peripheral blood of indicate treated Tc9 cells as Figure **2J** (n = 5). (**I-J**) $Thy1.1^+$ Pmel-1 Tc1 cells treated with RSL3 (0.05 μM) or Fer-1 (5 μM) were injected into B16 tumor-bearing $Thy1.2^+$ B6 mice with adjuvant treatments. Relative lipid ROS, $Thy1.1^+$ percentages in $CD8^+$ T and $Thy1.1^+$ $CD8^+$ T numbers of indicated Tc1 cells in tumor tissues on day 40 after tumor cell injection (n = 5). Data are presented as mean ± SEM. *$p < 0.05$; **$p < 0.01$. ***$p < 0.001$ with 1-way ANOVA followed by Dunnett's test in **H**, **I**, **J**, 2-way ANOVA in **F** and unpaired 2-tailed Student's *t* test in other panels.

Supplementary Figure 4. Positive correlation between ferroptosis and dysfunction in human melanoma $CD8^+$ T cells.

(**A**) Kaplan–Meier analysis (log-rank test) of overall survival in *IL9* high or *IL9* low melanoma, colon cancer and breast cancer patients with high $CD8^+$ infiltrates (see Method). (**B-C**) Melanoma patients' tumor-infiltrating $CD8^+$ T cells were divided into dysfunctional and non-dysfunctional groups from previous study. GSEA and heatmap analyses of lipid peroxidation and ferroptosis activation-related genes in these different groups. (**D-E**) Melanoma patients were divided into non-responders and responders who received PD-1 or CTLA-4 checkpoint immunotherapy by analyzing previous data. GSEA and heatmap analyses of lipid peroxidation and ferroptosis activation-related genes in non-responders and responders.

Supplementary Figure 5. Tc9 cells display increased fatty acid oxidation activity compared to Tc1 cells.

(**A-C**) Relative lipid ROS, iron level, and relative cell viability in Tc1 and Tc9 treated with FFA or AA for 16 hours on day 4 of polarization (n = 6). (**D**) Heatmap analysis of fatty acid synthesis- and uptake- related genes in tumor-infiltrating Tc1 or Tc9 cells on day 14 after injection. (**E-F**) Relative mRNA expression of fatty acid β-oxidation-, synthesis- and uptake- related genes (n = 3). (**G**) Cpt1a expression in polarized Tc1 or Tc9 cells from WT mice on day 4 of polarization (n = 3). Data are presented as mean ± SEM. *p < 0.05; ***p < 0.001 with 1-way ANOVA followed by Dunnett's test in **A**, **B**, **C,** and unpaired 2-tailed Student's *t* test in other panels.

Supplementary Figure 6. Increased fatty acid oxidation and mitochondrial activity are required for reduced lipid peroxidation and ferroptosis in Tc9 cells.

(**A**) MitoTracker intensity in polarized Tc1 and Tc9 cells (n = 6). (**B-C**) ECAR values at indicated treatments and basal OCR/ECAR ratio in Tc1 and Tc9 cells. (n = 6-8). (**D**) $TMRM^{high}$ ratio and intensity in tumor-infiltrating Tc1 and Tc9 cells from s.c. B16 tumor-bearing mice (n=10). (**E**) TMRM intensity in Tc1 and Tc9 cells from spleens of s.c. B16 tumor-bearing mice (n=10). (**F**) CPT1A expression in scramble- and *Cpt1a* shRNA lentivirus-transduced Tc9 cells. or vector- and *Cpt1a* overexpressing (OE) retrovirus-transduced Tc9 cells in **N** (n = 2). (**G**) TMRM intensity, relative lipid ROS and cell viability in scramble shRNA- or Cpt1a shRNA lentivirus-transduced Tc9 cells treated with RSL3 (0.05 μM) or TBH (60 μM) for 16h. (**H**) CPT1A expression vector- and *Cpt1a* overexpressing (OE) retrovirus-transduced Tc9 cells (n = 2). FFA: free fatty acids, AA: arachidoinic acid. Data are presented as mean ± SEM. *p < 0.05; ***p < 0.001 with two-tailed unpaired t test.

Supplementary Figure 7. IL9/STAT3 signaling induces Cpt1a expression in Tc9 cells.

(**A**) IPA result of JAK/Stat pathway in tumor-infiltrating Tc1 and Tc9 cells on day 14 after injection. Red: upregulation. (**B**) Cpt1a expression in Stattic-treated Tc9 cells on day 4 of polarization. (**C**) p-STAT3 and Cpt1a expression in polarized Tc9 cells treated with IL-6 (30 nM) for 16 hours (n = 4). (**D**) Relative *Il6*, *Il10*, *Il27* and *Il9* mRNA expression in Tc1 and Tc9 cells on day 4 of polarization (n = 3). (**E**) IPA result of the crosstalk of STAT3 pathway and IL-9 signaling in tumor-infiltrating Tc9 cells. Data are presented as mean ± SEM. *p < 0.05; ***p < 0.001 with 1-way ANOVA followed by Dunnett's test in **B** and unpaired 2-tailed Student's *t* test in other panels.

Supplementary Figure 8. IL-9 signaling up-regulates fatty acid oxidation to resist tumor or ROS induced ferroptosis in Tc9 cells.

(**A**) LC-MS analysis of indicated saturated, mono-unsaturated, and poly-unsaturated fatty acid contents in naïve $CD8^+$ T cells isolated from WT or $Il9^{-/-}$ mice before polarization (n=2). (**B**) Cpt1a expression in naïve $CD8^+$ T cells in **A** (n=2). (**C**) LC-MS analysis of indicated saturated, mono-unsaturated, and poly-unsaturated fatty acid contents in polarized Tc1 or Tc9 from WT or $Il9^{-/-}$ mice on day 4 of polarization (n=2). (**D-G**) TMRM intensity, cellular ROS level, relative lipid ROS, iron level and relative cell viability in polarized Tc9 cells from indicated mice treated with ROS inducer TBH for 16 hours (n = 3). (**H-J**) TMRM intensity, cellular ROS level, relative lipid ROS, iron level and relative cell viability in vector- or Cpt1a overexpression transduced Tc9 cells from indicated mice treated with ROS inducer TBH for 16 hours (n = 3). (**K-M**) TMRM intensity, cellular ROS level, relative lipid ROS, iron level and relative cell viability in vector- or Cpt1a overexpression transduced Tc9 cells from indicated mice after co-culture with B16 cells for 48 hours (n = 3). Data are presented as mean ± SEM. *p < 0.05; **p < 0.01; ***p < 0.001 with 1-way ANOVA followed by Dunnett's test.

Figure 9. IL9 signaling deficiency dampens longevity of Tc9 cells in peripheral blood.

Thy1.1$^+$ Pmel-1 Tc9 cells from Pmel-1-WT, -*Il9*$^{-/-}$ and -*Il9r*$^{-/-}$ mice were injected into Thy1.2$^+$ B6 mice bearing 10-day s.c. B16 tumor as Figure **6A**. **(A)**Relative lipid ROS, **(B)** iron level, **(C)** TMRM intensity**, (D)** Thy1.1$^+$ percentages in CD8$^+$ T cells of transferred T cells in peripheral blood (n = 5). Data are presented as mean ± SEM. *p < 0.05; **p < 0.01 with 1-way ANOVA followed by Dunnett's test.

Figure 10. Inhibiting STAT3-fatty acid oxidation pathway in Tc9 cells impairs their longevity in peripheral blood.

(**A-E**) Thy1.1$^+$ Pmel-1 Tc9 cells were treated with DMSO (Ctrl), p-STAT3 inhibitor Stattic (0.5 μM), fatty acid oxidation inhibitor etomoxir (Eto, 50 μM), or ferroptosis inhibitor Fer-1 (5 μM) and then injected into Thy1.2$^+$ B6 mice bearing 10-day s.c. B16 tumor as Figure **7A**. Relative lipid ROS, iron level, TMRM intensity**,** Thy1.1$^+$ percentages in CD8$^+$ T cells of transferred T cells in peripheral blood (n = 5-6). (**F-H**) Thy1.1$^+$ Pmel-1 Tc9 cells were overexpressed with vector or *Cpt1a* and injected into Thy1.2$^+$ B6 mice bearing 10-day s.c. B16 tumor as Figure **7H**. Relative lipid ROS, TMRM intensity, Thy1.1$^+$ percentages in CD8$^+$ T cells of transferred T cells in peripheral blood (n = 6). (**I-M**) Thy1.1$^+$ Pmel-1 Tc1 or Tc9 cells from WT mice transduced with vector or Cpt1a were injected into B16 tumor bearing Thy1.2$^+$ B6 mice with adjuvant treatments. Relative lipid ROS, TMRM intensity, Thy1.1$^+$ percentages in CD8$^+$ T cells and Thy1.1$^+$ CD8$^+$ T cells numbers, tumor growth curve, and the expression of PD-1 and LAG-3 on transferred CD8$^+$ Thy1.1$^+$ T cells in tumors (n = 5). (**N**) Schematic diagram shows that IL/9/STAT3/fatty acid oxidation mediated lower lipid peroxidation contributed to Tc9 cell longevity and enhanced antitumor activity. Eto: Etomoxir, OE: overexpressing. Data are presented as mean ± SEM. *p < 0.05; **p < 0.01; ***p < 0.001 with 1-way ANOVA followed by Dunnett's test in **A**, **B**, **E**, 1-way ANOVA followed by Turkey's test in **I, J, K, M**, 2-way ANOVA in **L** and unpaired 2-tailed Student's *t* test in other panels.

Supplementary Figure 1

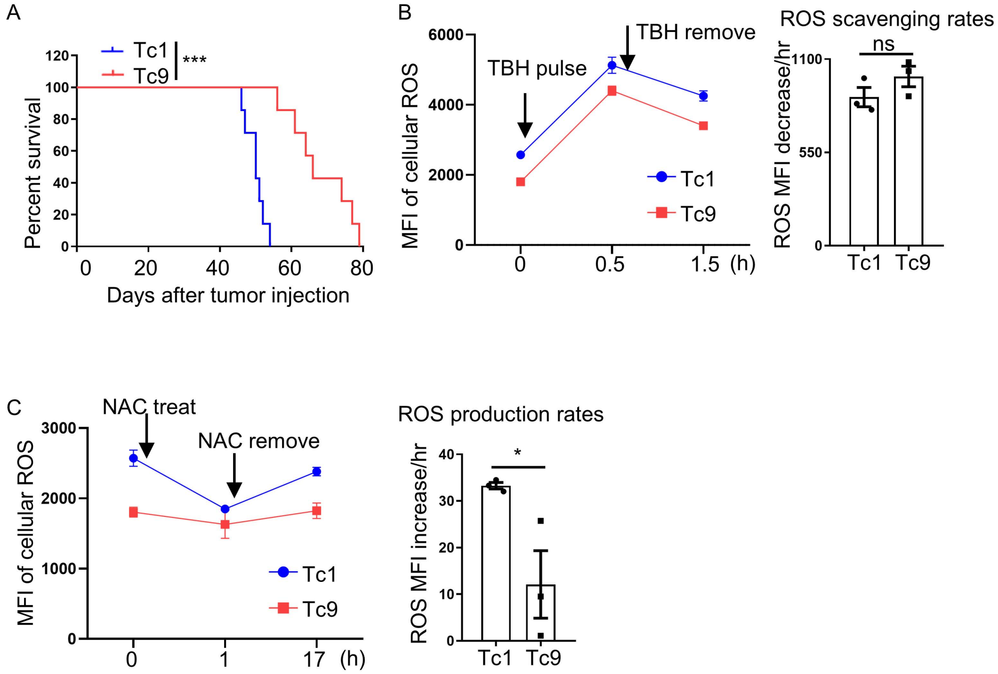

Supplementary Figure 2

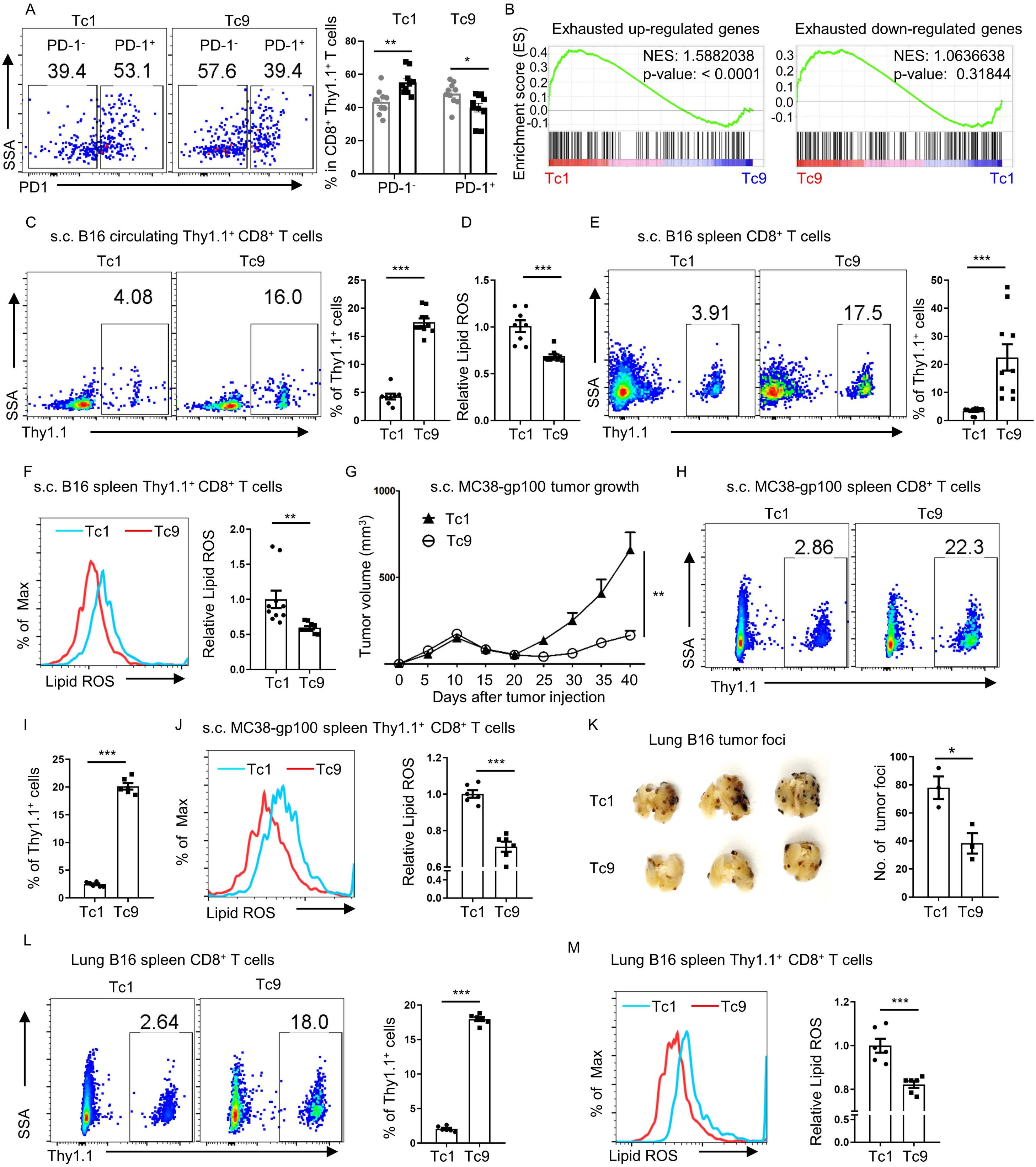

Supplementary Figure 3

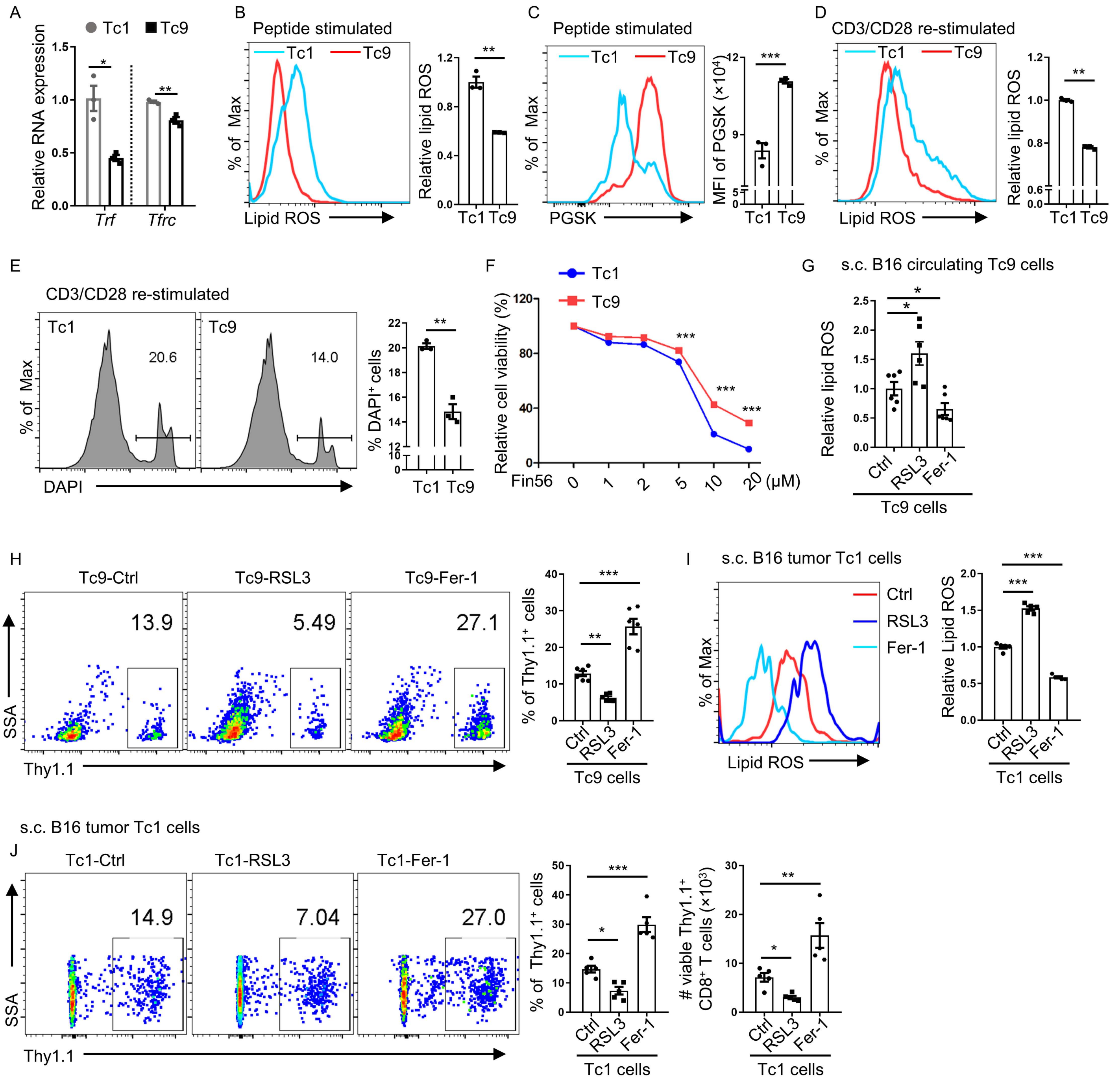

Supplementary Figure 4

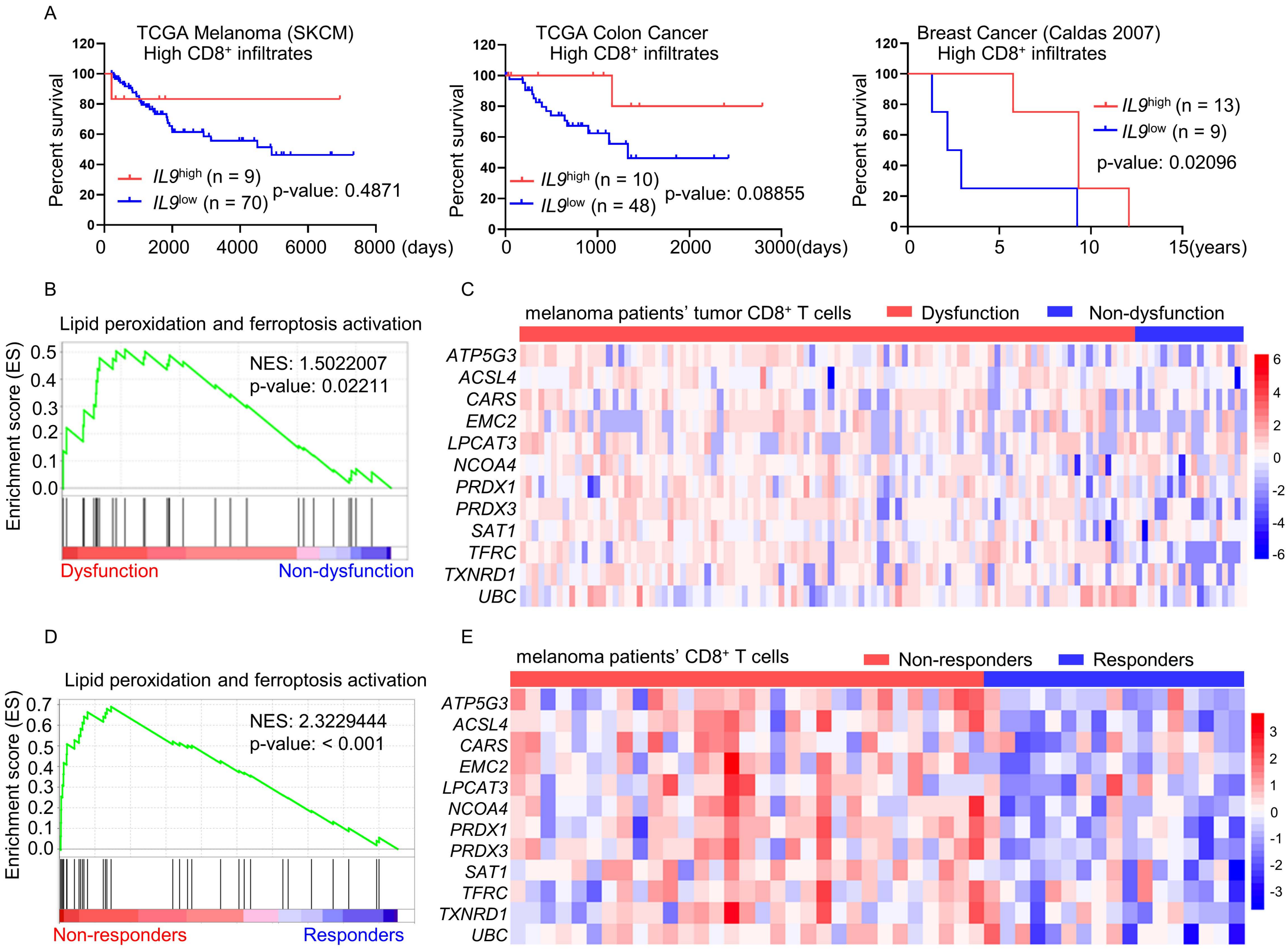

Supplementary Figure 5

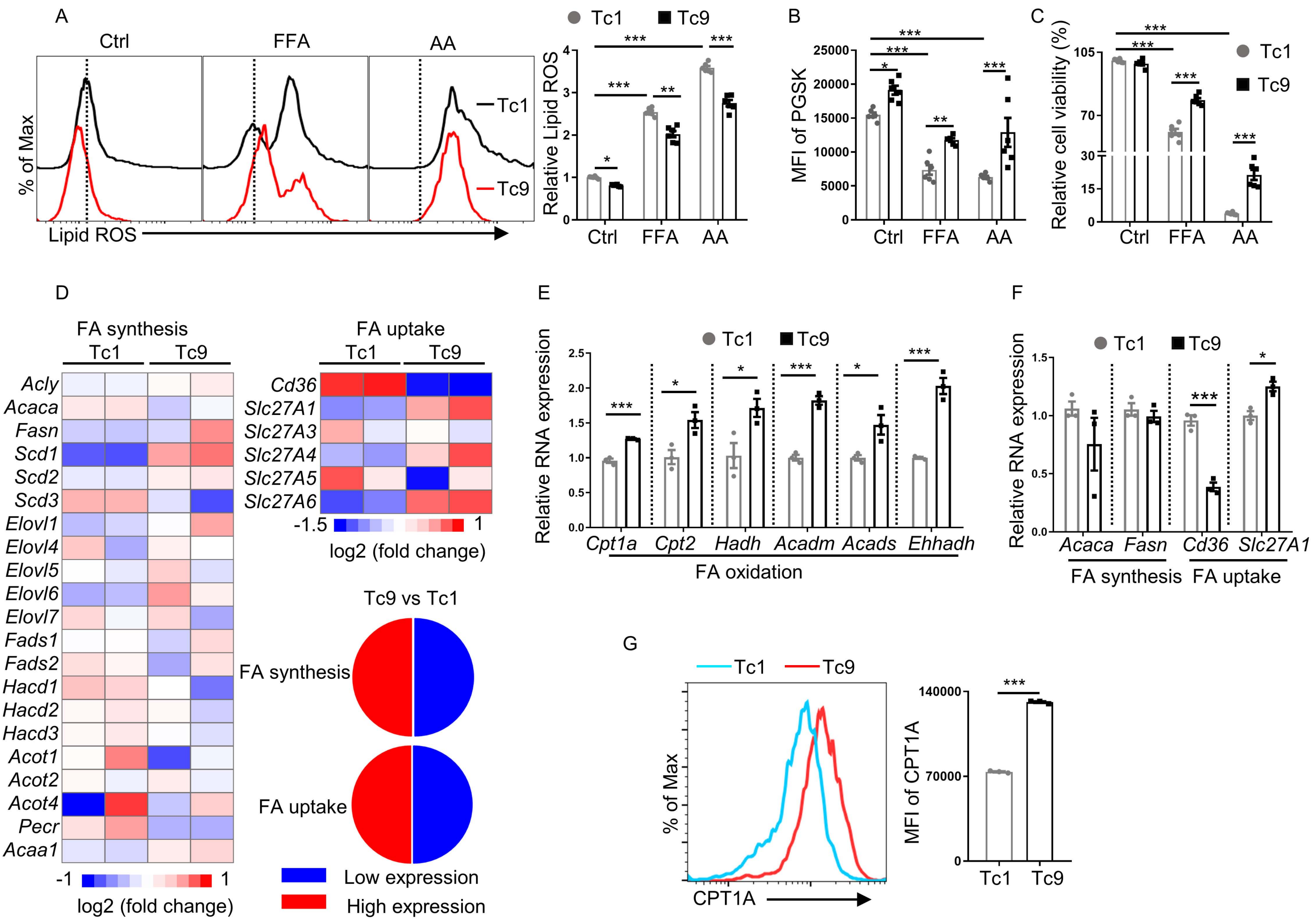

Supplementary Figure 6

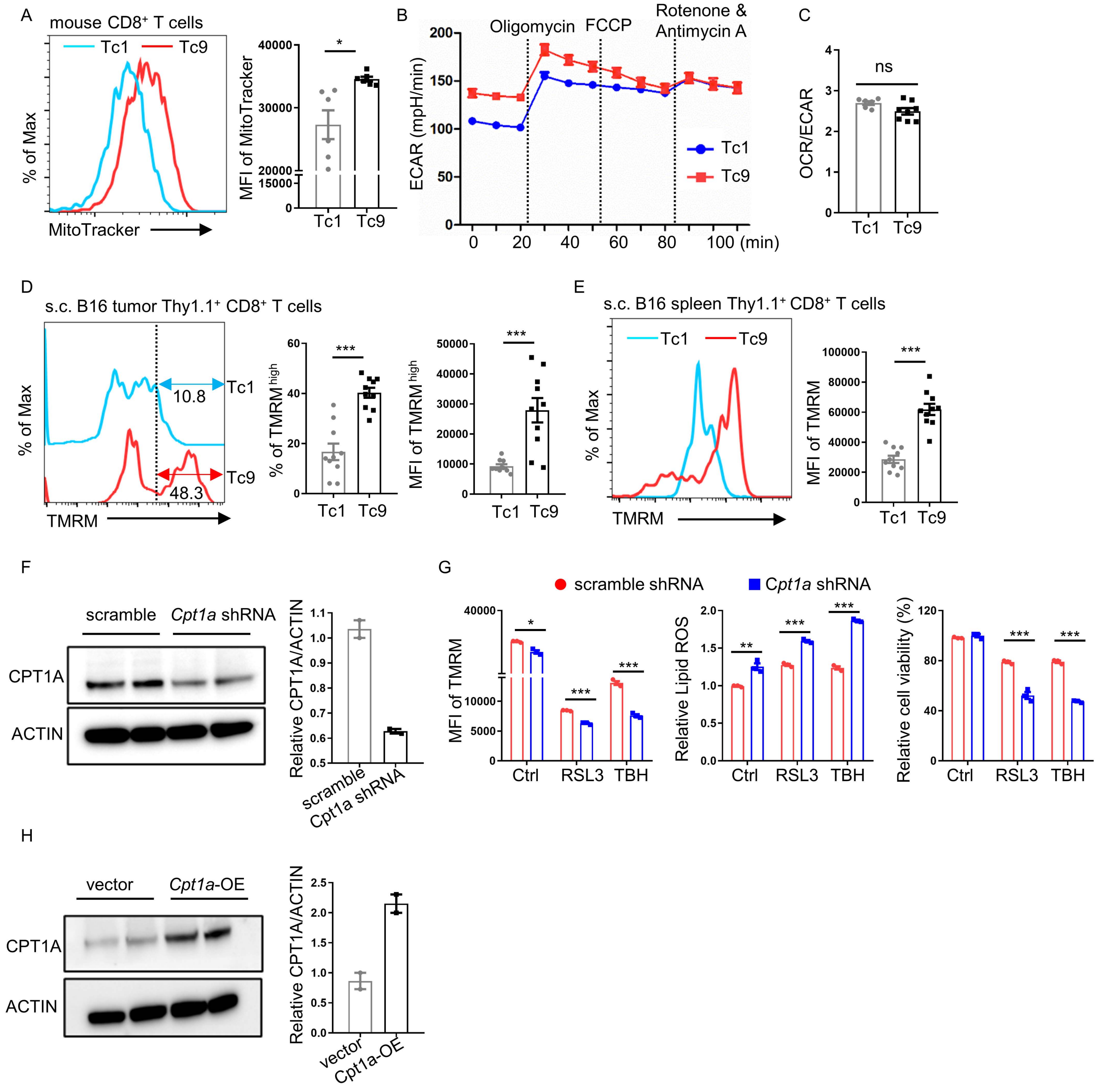

Supplementary Figure 7

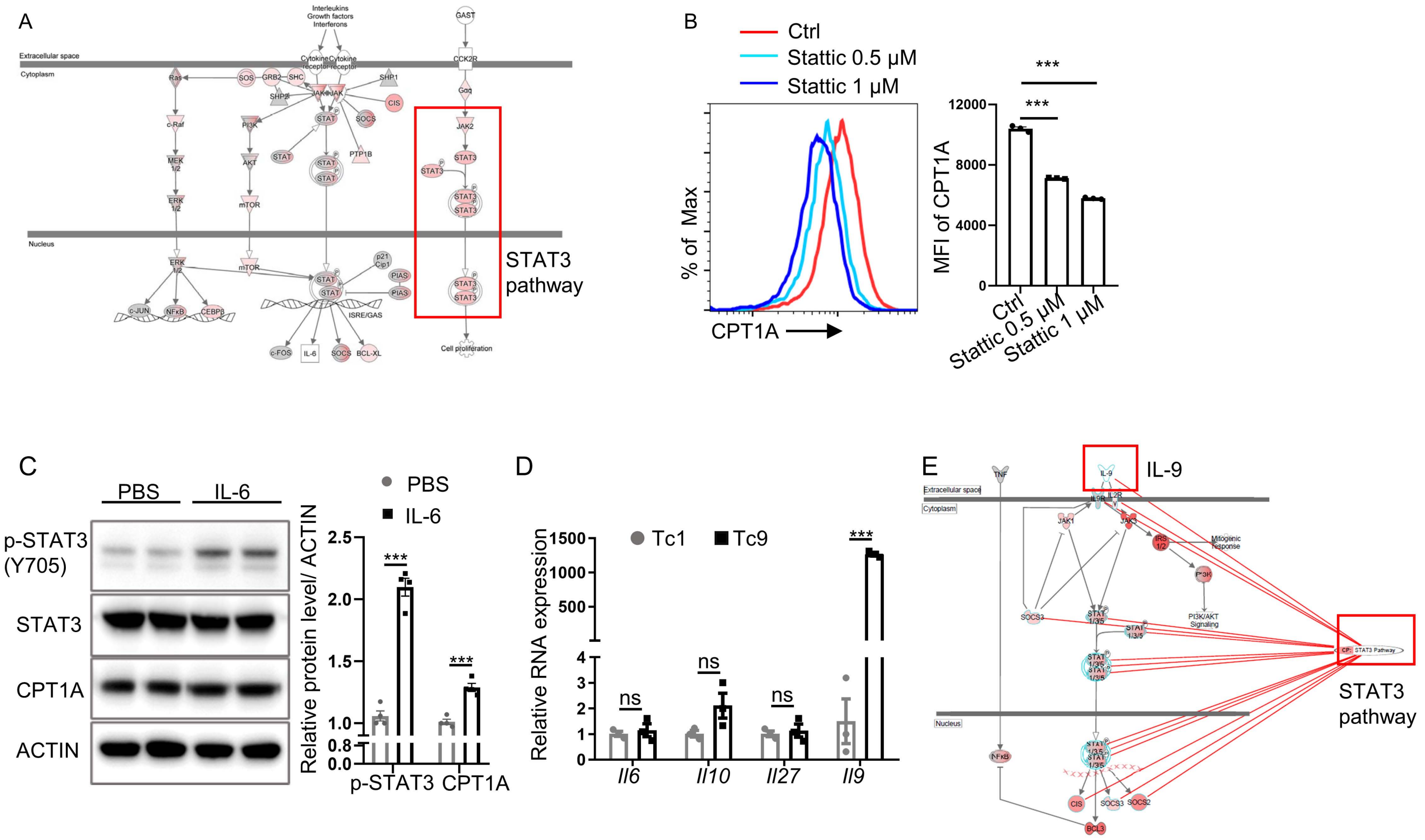

Supplementary Figure 8

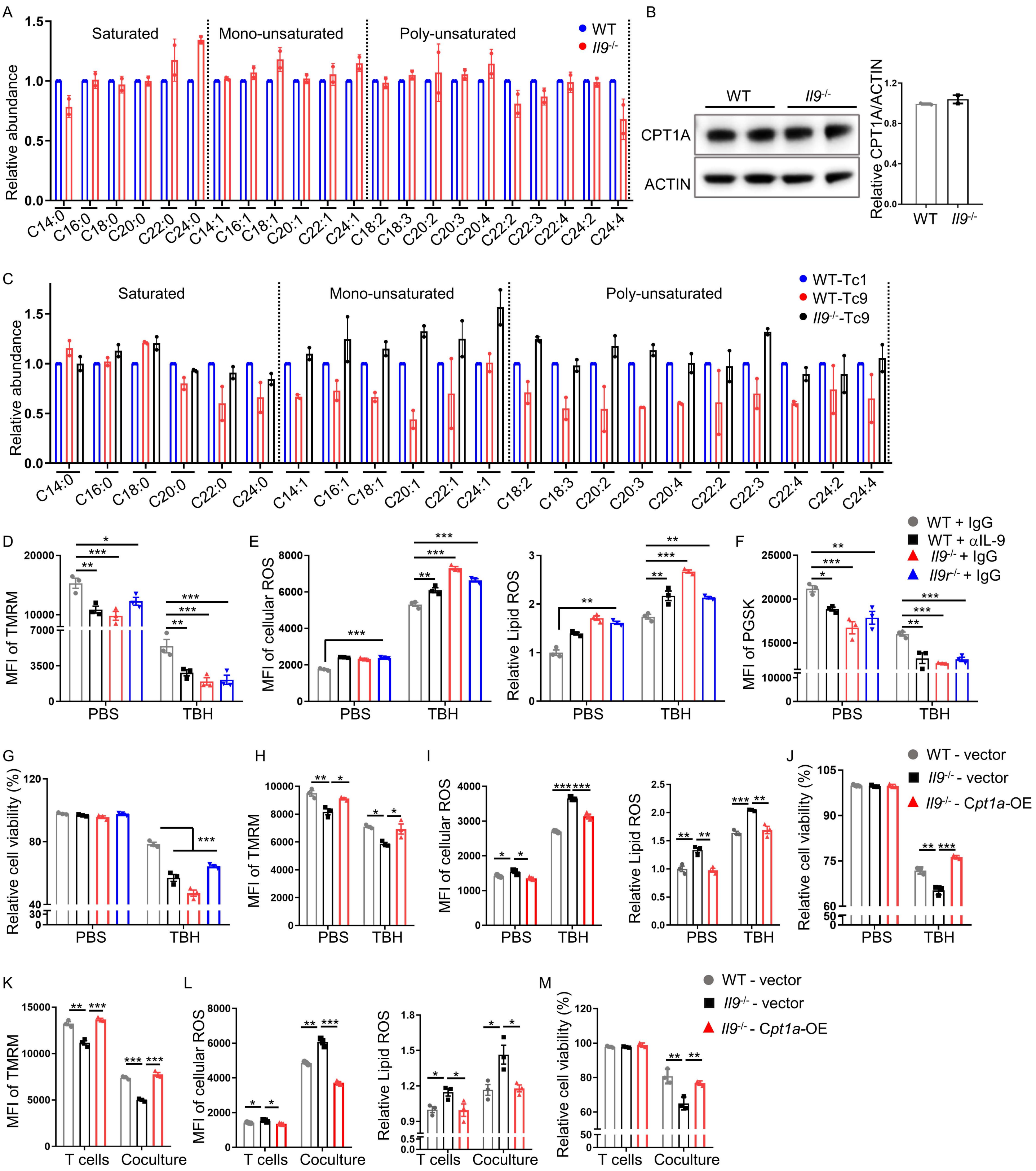

Supplementary Figure 9

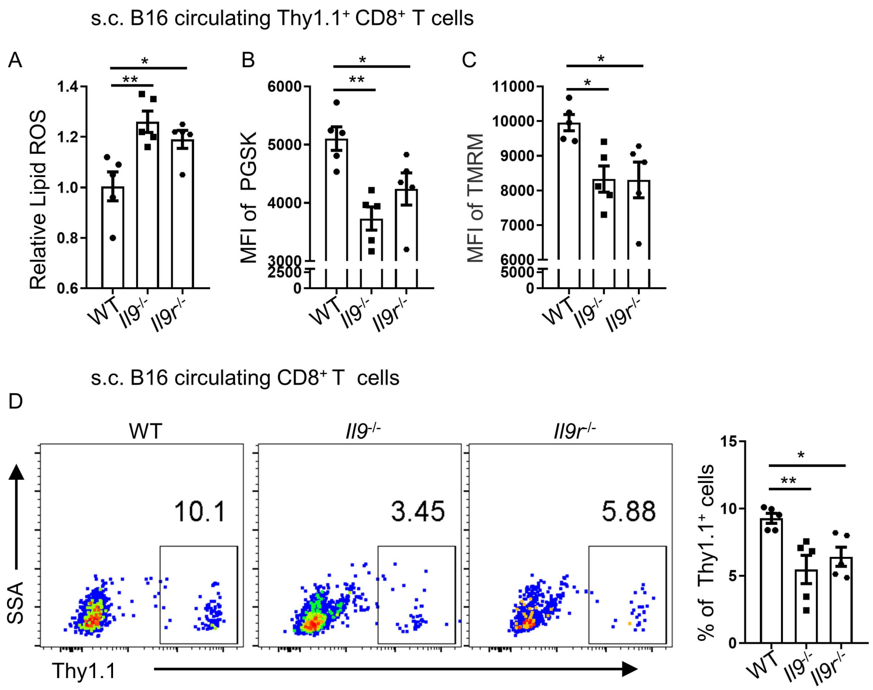

Supplementary Figure 10

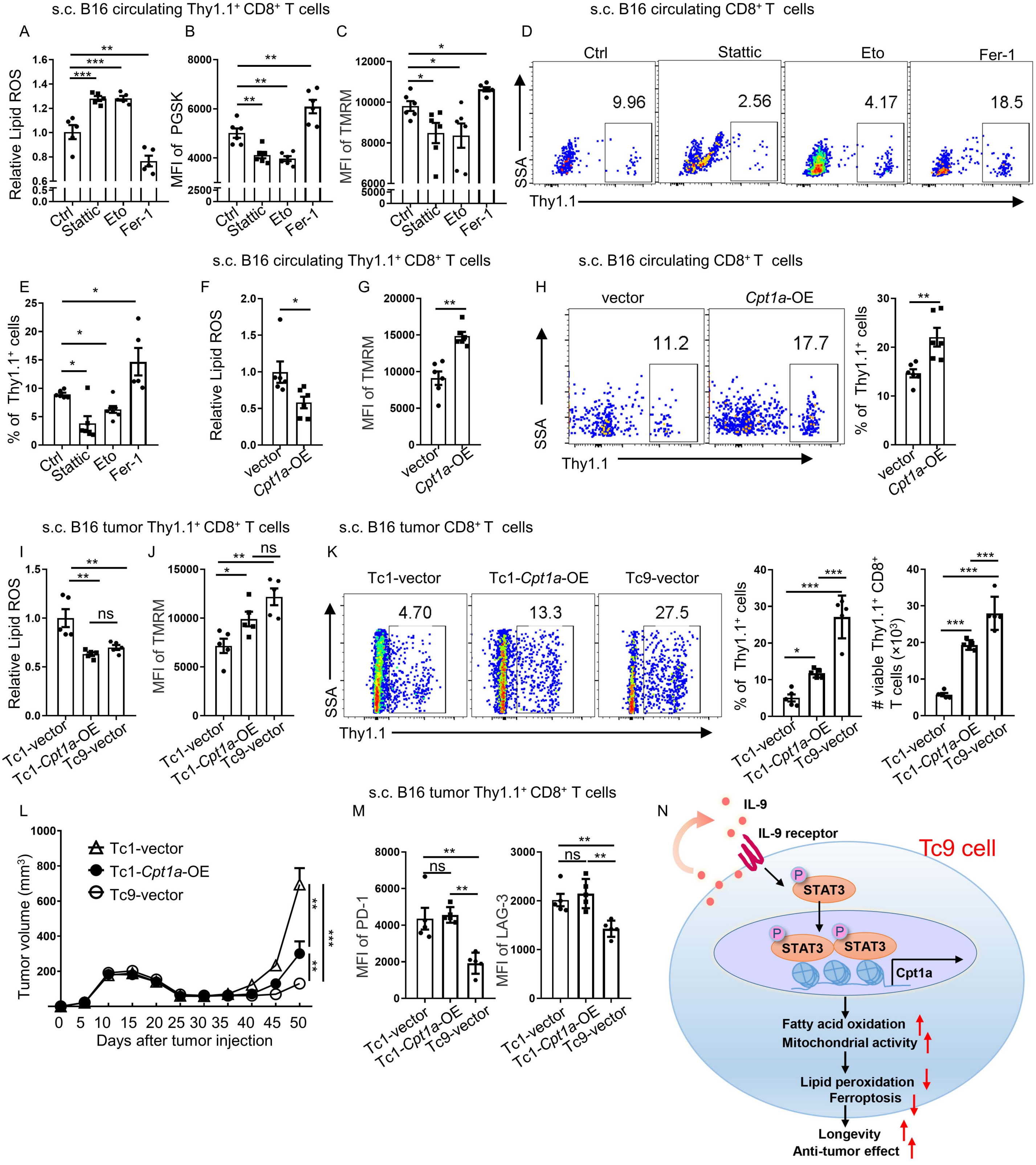

Supplement: Supplemental data [file jci-132-153247-s048.pdf]
