# Supplementary material for: IL-9/STAT3/fatty acid oxidation–mediated lipid peroxidation contributes to Tc9 cell longevity and enhanced antitumor activity
Source: J Clin Invest. 2022 Apr 1;132(7):e153247. doi: 10.1172/JCI153247 (PMC8970676; doi:10.1172/JCI153247)
Supplement: Supplemental table 1 [file jci-132-153247-s049.pdf]

Genes list of lipid peroxidation and ferroptosis activation related genes

ACSL4
ALOX12
ALOX5
ALOXE3
AOX1
ATP5G3
CARS
CHAC1
CS
DPP4
DUOX1
DUSP18
EMC2
GLS2
GUSB
HMBS
LPCAT3
MT3
NCF1
NCOA4
NOS2
PRDX1
PRDX3
PTGS2
PXDN
RNF7
RPL8
SAT1
SGK2
SRXN1
TFRC
TRP53
TXNRD1
TXNRD2
UBC

Student's t-test analysis of lipid peroxidation and ferroptosis activation related genes in $CD8^+$ T cells from melanoma patients' PBMC and tumors

obs.x	obs.y	obs.tot	mean.x	mean.y	mean.diff	var.x	var.y	var.pooled	stderr	df	statistic	pvalue	conf.low	conf.high	alternative	conf.level	gene
176	19	195	3.11446166	1.20137131	1.91309035	3.17521231	2.34076597	3.0973883	0.42499322	193	4.50146091	1.17E-05	1.07486277	2.75131793	two.sided	0.95	ACSL4
176	19	195	0.0682335	0.32635268	-0.2581192	0.10405409	2.02361535	0.28308053	0.12848108	193	-2.0090054	0.04592993	-0.5115265	-0.0047119	two.sided	0.95	ALOX12
176	19	195	0.82110064	0.7791077	0.04199294	2.11760798	2.1326636	2.11901213	0.35152069	193	0.1194608	0.90503455	-0.6513224	0.73530834	two.sided	0.95	ALOX5
176	19	195	0	0	0	0	0	0	0	193	NA	NA	NA	NA	two.sided	0.95	ALOXE3
176	19	195	0.02569985	0	0.02569985	0.11624487	0	0.10540338	0.07839917	193	0.32780768	0.74341241	-0.1289293	0.18032901	two.sided	0.95	AOX1
176	19	195	4.66811629	2.92322432	1.74489197	2.58982789	3.41667634	2.66694329	0.39435837	193	4.42463532	1.61E-05	0.96708646	2.52269747	two.sided	0.95	ATP5G3
176	19	195	2.55124435	1.62978019	0.92146416	2.91871994	3.21924093	2.9467478	0.4145297	193	2.22291468	0.0273821	0.1038741	1.73905423	two.sided	0.95	CARS
176	19	195	0.20790293	0	0.20790293	0.59329956	0	0.53796592	0.17711753	193	1.17381335	0.24191625	-0.1414316	0.55723745	two.sided	0.95	CHAC1
176	19	195	3.13228682	2.45684563	0.67544119	2.93049582	2.95590311	2.93286541	0.41355211	193	1.63326744	0.10404305	-0.1402207	1.49110311	two.sided	0.95	CS
176	19	195	1.43131712	1.61669175	-0.1853746	2.18611903	2.94671898	2.25705582	0.36279001	193	-0.5109695	0.6099564	-0.9009169	0.53016759	two.sided	0.95	DPP4
176	19	195	0.13053088	0.14338856	-0.0128577	0.31660676	0.39064532	0.32351191	0.1373502	193	-0.0936124	0.92551417	-0.2837578	0.25804247	two.sided	0.95	DUOX1
176	19	195	1.54189682	0.85790442	0.68399241	2.24021792	2.07396499	2.22471246	0.36018126	193	1.89902274	0.05905317	-0.0264045	1.39438932	two.sided	0.95	DUSP18
176	19	195	2.09925843	0.9866885	1.11256993	2.62632653	1.5213384	2.52327064	0.38358895	193	2.90042227	0.00415904	0.3560053	1.86913457	two.sided	0.95	EMC2
176	19	195	0.08903691	0.0703515	0.0186854	0.1628454	0.09403735	0.15642807	0.09550839	193	0.19564151	0.84509659	-0.1696888	0.20705962	two.sided	0.95	GLS2
176	19	195	2.70580576	0.63942032	2.06638544	2.88017431	1.62314318	2.76293825	0.40139297	193	5.14803597	6.46E-07	1.27470537	2.85806551	two.sided	0.95	GUSB
176	19	195	1.27654138	0.63903679	0.63750459	1.96735962	1.35950115	1.91066815	0.33379268	193	1.90988189	0.05763128	-0.0208453	1.29585446	two.sided	0.95	HMBS
176	19	195	1.8866532	0.71749189	1.1691613	2.32214757	1.64115518	2.25863533	0.36291693	193	3.22156726	0.00149642	0.45336874	1.88495386	two.sided	0.95	LPCAT3
176	19	195	0.35402774	0.29753533	0.05649241	0.71314936	0.5803917	0.70076782	0.20214884	193	0.27945948	0.78019148	-0.3422121	0.45519696	two.sided	0.95	MT3
176	19	195	0.58610171	0.32876006	0.25734165	1.14243082	0.62350303	1.09403341	0.25258029	193	1.01885086	0.30954924	-0.2408305	0.75551376	two.sided	0.95	NCF1
176	19	195	4.06608803	2.63561006	1.43047797	2.28622451	3.69304624	2.41743068	0.37545785	193	3.80995623	1.87E-04	0.68995056	2.17100537	two.sided	0.95	NCOA4
176	19	195	0.01522355	0	0.01522355	0.02051055	0	0.01859765	0.03293163	193	0.46227766	0.64440292	-0.0497285	0.08017564	two.sided	0.95	NOS2
176	19	195	3.92187549	2.43791954	1.48395595	2.7867939	3.29140381	2.83385596	0.40651171	193	3.650463	3.37E-04	0.68218003	2.28573186	two.sided	0.95	PRDX1
176	19	195	2.94140243	1.58362395	1.35777847	3.19571072	2.7615185	3.15521611	0.42894215	193	3.16541162	0.00179984	0.51176228	2.20379467	two.sided	0.95	PRDX3
176	19	195	0.30646406	0	0.30646406	0.89894173	0	0.8151026	0.21801698	193	1.40568896	0.16142428	-0.1235377	0.73646584	two.sided	0.95	PTGS2
176	19	195	0.28212528	0	0.28212528	0.64643134	0	0.58614241	0.18487822	193	1.52600604	0.12864543	-0.0825159	0.64676644	two.sided	0.95	PXDN
176	19	195	3.45941097	3.17518418	0.28422679	3.08524721	3.67243842	3.14001116	0.42790737	193	0.66422504	0.50733905	-0.5597485	1.12820205	two.sided	0.95	RNF7
176	19	195	6.98298946	7.06348646	-0.080497	0.1304286	0.17000197	0.13411938	0.08843617	193	-0.910227	0.36383815	-0.2549225	0.09392847	two.sided	0.95	RPL8
176	19	195	4.67786104	2.81469167	1.86316936	2.41751267	3.80412396	2.54683393	0.38537584	193	4.83468129	2.72E-06	1.10308039	2.62325834	two.sided	0.95	SAT1
176	19	195	0.02202087	0	0.02202087	0.04346713	0	0.0394132	0.04794077	193	0.45933488	0.64651064	-0.0725342	0.11657597	two.sided	0.95	SGK2
176	19	195	0	0	0	0	0	0	0	193	NA	NA	NA	NA	two.sided	0.95	SRXN1
176	19	195	2.75386602	1.1435705	1.61029552	3.08697173	2.24361948	3.00831711	0.4188379	193	3.84467475	1.64E-04	0.78420825	2.43638278	two.sided	0.95	TFRC
176	19	195	2.80379302	1.75681375	1.04697927	2.94673869	2.79161457	2.93227115	0.41351021	193	2.53193089	0.0121405	0.23139999	1.86255855	two.sided	0.95	TXNRD1
176	19	195	1.06443448	0.51562474	0.54880974	1.57122285	1.63483752	1.57715583	0.30326443	193	1.80967397	0.07190212	-0.0493283	1.14694778	two.sided	0.95	TXNRD2
176	19	195	6.13644557	5.14320354	0.99324204	0.86808682	1.78320338	0.95343448	0.23579229	193	4.21236014	3.88E-05	0.52818144	1.45830264	two.sided	0.95	UBC

x: tumor y: PBMC

Student's t-test analysis of lipid peroxidation and ferroptosis activation related genes in dysfunctinal and non-dysfunctional tumor-infitrating CD8$^+$ T cells from melanoma patients

obs.x	obs.y	obs.tot	mean.x	mean.y	mean.diff	var.x	var.y	var.pooled	stderr	df	statistic	pvalue	conf.low	conf.high	alternative	conf.level	gene
100	18	118	3.49017336	2.67911428	0.81105908	1.70016741	3.38479321	1.94705222	0.35726753	116	2.27017292	0.02504354	0.10344571	1.51867244	two.sided	0.95	ACSL4
100	18	118	0.0719417	0.16531695	-0.0933753	0.13158287	0.49193452	0.18439303	0.10994538	116	-0.8492877	0.39747059	-0.3111359	0.12438543	two.sided	0.95	ALOX12
100	18	118	0.19403092	0.24721962	-0.0531887	0.37218272	0.52121237	0.39402327	0.16071843	116	-0.3309434	0.74128432	-0.3715118	0.26513441	two.sided	0.95	ALOX5
100	18	118	0	0	0	0	0	0	0	116	NA	NA	NA	NA	two.sided	0.95	ALOXE3
100	18	118	0	0	0	0	0	0	0	116	NA	NA	NA	NA	two.sided	0.95	AOX1
100	18	118	5.22487001	4.74289851	0.4819715	0.46882436	1.70879642	0.6505444	0.20651102	116	2.33387791	0.02132346	0.07295041	0.89099259	two.sided	0.95	ATP5G3
100	18	118	3.06241163	2.10999787	0.95241376	2.26584508	3.14537929	2.39474234	0.39621795	116	2.40376227	0.01781124	0.16765418	1.73717333	two.sided	0.95	CARS
100	18	118	0.05761323	0	0.05761323	0.1729302	0	0.14758698	0.09836232	116	0.58572461	0.55919844	-0.1372057	0.2524322	two.sided	0.95	CHAC1
100	18	118	3.76708151	3.22259716	0.54448436	1.05590385	2.7471842	1.3037639	0.29235076	116	1.86243524	0.06507197	-0.0345532	1.12352187	two.sided	0.95	CS
100	18	118	1.22979783	0.92005163	0.3097462	1.99845304	2.51814863	2.07461533	0.36878525	116	0.83990942	0.40268694	-0.4206795	1.04017186	two.sided	0.95	DPP4
100	18	118	0.11294109	0	0.11294109	0.23968127	0	0.20455556	0.11580049	116	0.97530749	0.33143727	-0.1164164	0.34229855	two.sided	0.95	DUOX1
100	18	118	1.60927543	1.10998383	0.4992916	2.32186806	2.12202449	2.29258064	0.38767434	116	1.28791498	0.20033888	-0.2685463	1.26712948	two.sided	0.95	DUSP18
100	18	118	2.30074515	1.16014927	1.14059589	2.32038341	3.0087136	2.42125939	0.39840558	116	2.8629014	0.00498263	0.35150344	1.92968833	two.sided	0.95	EMC2
100	18	118	0.08985626	0	0.08985626	0.15540638	0	0.13263131	0.09324548	116	0.96365271	0.33722467	-0.0948282	0.27454067	two.sided	0.95	GLS2
100	18	118	3.05635885	2.49694209	0.55941675	1.39366392	2.07222536	1.49310827	0.31286026	116	1.78807227	0.07637505	-0.0602424	1.17907592	two.sided	0.95	GUSB
100	18	118	1.37053719	0.54108623	0.82945095	2.02010858	1.60309979	1.95899522	0.35836158	116	2.31456441	0.02239614	0.11967069	1.53923121	two.sided	0.95	HMBS
100	18	118	2.34575545	2.64431436	-0.2985589	2.20869923	2.46138424	2.24573065	0.38369274	116	-0.7781198	0.43808265	-1.0585107	0.46139291	two.sided	0.95	LPCAT3
100	18	118	0.45780084	0.19899602	0.25880482	1.08764221	0.71278948	1.03270689	0.26019161	116	0.99467012	0.32196708	-0.2565374	0.77414709	two.sided	0.95	MT3
100	18	118	0.27168063	0.24721962	0.02446101	0.48750527	0.52121237	0.4924451	0.17967335	116	0.13614159	0.89194534	-0.3314047	0.38032672	two.sided	0.95	NCF1
100	18	118	4.30407487	4.1582876	0.14578727	0.75344106	1.6433852	0.88386391	0.24071184	116	0.60565061	0.54592998	-0.3309729	0.6225474	two.sided	0.95	NCOA4
100	18	118	0	0	0	0	0	0	0	116	NA	NA	NA	NA	two.sided	0.95	NOS2
100	18	118	4.44957553	4.23005631	0.21951922	0.25894854	0.43021027	0.28404724	0.13645833	116	1.60869056	0.11040233	-0.0507537	0.48979213	two.sided	0.95	PRDX1
100	18	118	3.80178427	2.34198994	1.45979433	1.27251574	3.24269672	1.56124916	0.31991961	116	4.56300361	1.26E-05	0.82615323	2.09343543	two.sided	0.95	PRDX3
100	18	118	0.13452806	0.26750336	-0.1329753	0.32097597	0.69182918	0.37532515	0.15685869	116	-0.8477394	0.39832888	-0.4436537	0.1777031	two.sided	0.95	PTGS2
100	18	118	0.25247333	0.16774092	0.08473241	0.56695528	0.50646629	0.55809051	0.1912745	116	0.44298855	0.65859904	-0.2941108	0.46357566	two.sided	0.95	PXDN
100	18	118	4.27124618	3.99417487	0.27707131	0.61365219	1.38448918	0.72661968	0.21825205	116	1.26950153	0.20680326	-0.1552044	0.70934699	two.sided	0.95	RNF7
100	18	118	6.87044439	6.98550498	-0.1150606	0.06389858	0.08618611	0.06716486	0.06635533	116	-1.7340067	0.08557359	-0.2464857	0.0163645	two.sided	0.95	RPL8
100	18	118	4.47272784	4.39602737	0.07670047	0.53936984	1.44938822	0.6727346	0.21000355	116	0.36523417	0.71560171	-0.339238	0.49263896	two.sided	0.95	SAT1
100	18	118	0.02334144	0	0.02334144	0.05448227	0	0.0464978	0.05521041	116	0.42277242	0.673244	-0.0860097	0.13269261	two.sided	0.95	SGK2
100	18	118	0	0	0	0	0	0	0	116	NA	NA	NA	NA	two.sided	0.95	SRXN1
100	18	118	3.57886844	1.39440766	2.18446079	1.42205959	2.65253183	1.60238742	0.32410708	116	6.73993547	6.47E-10	1.54252588	2.82639569	two.sided	0.95	TFRC
100	18	118	3.19605194	2.39961578	0.79643616	1.73519709	3.46506687	1.98871249	0.36106946	116	2.20576995	0.02937122	0.0812926	1.51157971	two.sided	0.95	TXNRD1
100	18	118	1.21696911	1.58017615	-0.363207	1.84857285	2.90287245	2.00308228	0.36237159	116	-1.0023055	0.31828231	-1.0809296	0.35451557	two.sided	0.95	TXNRD2
100	18	118	6.3035372	6.12818377	0.17535343	0.05708253	0.07210701	0.05928439	0.06234117	116	2.81280308	0.00576931	0.05187889	0.29882797	two.sided	0.95	UBC

x: Dysfucntion   y: Non-dysfunction

Student's t-test analysis of lipid peroxidation and ferroptosis activation related genes in tumor-infitrating $CD8^+$ T cells from checkpoint immunotherapy responders and non-responders

obs.x	obs.y	obs.tot	mean.x	mean.y	mean.diff	var.x	var.y	var.pooled	stderr	df	statistic	pvalue	conf.low	conf.high	alternative	conf.level	gene
31	17	48	0.0043405	0.00303961	0.00130089	1.05E-04	3.48E-05	8.08E-05	0.00271313	46	0.47948046	0.63386871	-0.0041603	0.00676213	two.sided	0.95	NOS2
31	17	48	0.17237235	0.11350971	0.05886265	0.06118357	0.00931071	0.04314084	0.06268442	46	0.93903154	0.35261936	-0.0673145	0.18503983	two.sided	0.95	ALOX5
31	17	48	1.38165158	1.22438031	0.15727126	0.04491494	0.04746671	0.04580251	0.06458921	46	2.4349465	0.01883089	0.02725993	0.2872826	two.sided	0.95	CS
31	17	48	0.69916163	0.55826073	0.14090089	0.05636797	0.03224773	0.04797832	0.06610554	46	2.13145376	0.0384265	0.00783735	0.27396444	two.sided	0.95	ACSL4
31	17	48	1.46718333	1.33951368	0.12766965	0.05546446	0.08750934	0.06661051	0.07789089	46	1.63908322	0.10801845	-0.0291166	0.28445589	two.sided	0.95	TFRC
31	17	48	0.04147841	0.01213493	0.02934348	0.00251014	5.40E-04	0.00182499	0.01289276	46	2.27596562	0.02755057	0.0033917	0.05529527	two.sided	0.95	PTGS2
31	17	48	0.03480189	0.00851951	0.02628238	0.00378021	6.24E-04	0.0026823	0.01563036	46	1.68149531	0.09944496	-0.0051799	0.05774467	two.sided	0.95	MT3
31	17	48	0.00638589	0.01160485	-0.005219	2.30E-04	4.15E-04	2.95E-04	0.00517961	46	-1.007596	0.31891944	-0.015645	0.00520706	two.sided	0.95	SGK2
31	17	48	0.642006	0.42665903	0.21534698	0.05557316	0.02595729	0.04527199	0.06421406	46	3.35358004	0.00160449	0.08609079	0.34460317	two.sided	0.95	EMC2
31	17	48	0.05331629	0.0542368	-9.21E-04	0.00221047	0.00442828	0.00298188	0.01648012	46	-0.055856	0.95569845	-0.0340933	0.03225225	two.sided	0.95	ALOX12
31	17	48	0.84665975	0.74729233	0.09936741	0.0387592	0.04753594	0.04181198	0.06171144	46	1.61019441	0.11419732	-0.0248513	0.2235861	two.sided	0.95	CARS
31	17	48	0.79249014	0.65769694	0.1347932	0.02721661	0.03959602	0.03152249	0.05358282	46	2.51560479	0.01543799	0.02693658	0.24264982	two.sided	0.95	LPCAT3
31	17	48	1.29918391	1.08853855	0.21064536	0.03278929	0.01377652	0.02617615	0.04882792	46	4.31403501	8.41E-05	0.11235985	0.30893086	two.sided	0.95	RNF7
31	17	48	1.61431793	1.36383513	0.2504828	0.04719654	0.05273805	0.04912402	0.06689016	46	3.74468813	5.01E-04	0.11583989	0.38512572	two.sided	0.95	PRDX1
31	17	48	0.05034128	0.0663481	-0.0160068	0.00346297	0.00770113	0.00493711	0.02120566	46	-0.7548376	0.45419387	-0.0586916	0.02667795	two.sided	0.95	CHAC1
31	17	48	2.14204385	1.93310808	0.20893578	0.01818924	0.05347986	0.03046424	0.05267572	46	3.96645333	2.53E-04	0.10290506	0.3149665	two.sided	0.95	SAT1
31	17	48	0.04871307	0.03119496	0.01751812	0.00801937	0.00210456	0.00596204	0.02330305	46	0.75175201	0.4560295	-0.0293885	0.06442473	two.sided	0.95	PXDN
31	17	48	0.10650787	0.14926687	-0.042759	0.0066001	0.013046	0.00884216	0.02837882	46	-1.506722	0.13871909	-0.0998826	0.01436461	two.sided	0.95	GLS2
31	17	48	0.20042439	0.24972905	-0.0493047	0.01291452	0.0135203	0.01312523	0.03457552	46	-1.425999	0.16062014	-0.1189016	0.02029225	two.sided	0.95	DUOX1
31	17	48	1.15668596	0.9220372	0.23464876	0.04633654	0.03358997	0.04190295	0.06177853	46	3.79822489	4.26E-04	0.11029503	0.3590025	two.sided	0.95	NCOA4
31	17	48	0.00441123	0.00610711	-0.0016959	1.30E-04	2.25E-04	1.63E-04	0.0038573	46	-0.4396553	0.66224549	-0.0094602	0.00606845	two.sided	0.95	AOX1
31	17	48	2.44012994	2.36612439	0.07400556	0.00230458	0.01787794	0.0077214	0.02651938	46	2.79062137	0.00763269	0.02062481	0.1273863	two.sided	0.95	UBC
31	17	48	1.57930389	1.2470942	0.33220968	0.06049852	0.06234578	0.06114105	0.07462455	46	4.45174762	5.39E-05	0.18199825	0.48242112	two.sided	0.95	ATP5G3
31	17	48	0.37242133	0.37989265	-0.0074713	0.04623591	0.03362646	0.04185001	0.0617395	46	-0.1210136	0.9042073	-0.1317465	0.11680385	two.sided	0.95	NCF1
31	17	48	2.39106982	2.3748357	0.01623412	0.00287787	0.00754254	0.00450036	0.02024599	46	0.80184375	0.42676633	-0.0245189	0.05698719	two.sided	0.95	RPL8
31	17	48	1.25405056	0.86573476	0.3883158	0.10381874	0.06482997	0.09025743	0.09066858	46	4.2828047	9.30E-05	0.20580942	0.57082217	two.sided	0.95	PRDX3
31	17	48	0.40477292	0.34438638	0.06038654	0.01859793	0.01408223	0.01702725	0.03938108	46	1.53338986	0.13202945	-0.0188835	0.13965653	two.sided	0.95	DUSP18
31	17	48	0.96910947	0.71671468	0.25239479	0.03996918	0.03048262	0.03666951	0.05779202	46	4.36729501	7.08E-05	0.13606549	0.36872408	two.sided	0.95	GUSB
31	17	48	0.00627356	0.00494283	0.00133073	1.71E-04	1.61E-04	1.68E-04	0.00390784	46	0.34052848	0.73500889	-0.0065353	0.00919679	two.sided	0.95	ALOXE3
31	17	48	0.32078444	0.28508335	0.03570109	0.03691857	0.01533523	0.02941132	0.05175742	46	0.68977724	0.49379962	-0.0684812	0.13988336	two.sided	0.95	TXNRD2
31	17	48	0.31229383	0.25983617	0.05245766	0.03673136	0.04337468	0.03904208	0.05963233	46	0.87968491	0.38360263	-0.067576	0.17249132	two.sided	0.95	DPP4
31	17	48	0.74939286	0.59044634	0.15894652	0.05561518	0.0407429	0.05044221	0.06778169	46	2.34497728	0.02339851	0.02250906	0.29538398	two.sided	0.95	TXNRD1
31	17	48	0.46166354	0.24039319	0.22127036	0.06067003	0.02094735	0.04685344	0.065326	46	3.38717136	0.00145537	0.08977594	0.35276478	two.sided	0.95	HMBS
31	17	48	0.09281734	0.08186114	0.0109562	0.00740125	0.00797572	0.00760107	0.02631193	46	0.41639657	0.67905639	-0.042007	0.06391936	two.sided	0.95	SRXN1

x: Non-responders y: Responders
